# The *Sinorhizobium meliloti *RNA chaperone Hfq influences central carbon metabolism and the symbiotic interaction with alfalfa

**DOI:** 10.1186/1471-2180-10-71

**Published:** 2010-03-06

**Authors:** Omar Torres-Quesada, Roke I Oruezabal, Alexandra Peregrina, Edgardo Jofré, Javier Lloret, Rafael Rivilla, Nicolás Toro, José I Jiménez-Zurdo

**Affiliations:** 1 Grupo de Ecología Genética de la Rizosfera, Estación Experimental del Zaidín, CSIC, Profesor Albareda, 1, 18008 Granada, Spain; 2 Departamento de Biología, Universidad Autónoma de Madrid, Campus de Cantoblanco, 28049 Madrid, Spain; 3 Departamento de Ciencias Naturales, Facultad de Ciencias Exactas, Físico-Químicas y Naturales, Universidad Nacional de Río Cuarto, Ruta 36 Km 601, X5804BYA Río Cuarto, Córdoba, Argentina

## Abstract

**Background:**

The bacterial Hfq protein is able to interact with diverse RNA molecules, including regulatory small non-coding RNAs (sRNAs), and thus it is recognized as a global post-transcriptional regulator of gene expression. Loss of Hfq has an extensive impact in bacterial physiology which in several animal pathogens influences virulence. *Sinorhizobium meliloti *is a model soil bacterium known for its ability to establish a beneficial nitrogen-fixing intracellular symbiosis with alfalfa. Despite the predicted general involvement of Hfq in the establishment of successful bacteria-eukaryote interactions, its function in *S. meliloti *has remained unexplored.

**Results:**

Two independent *S. meliloti *mutants, 2011-3.4 and 1021Δ*hfq*, were obtained by disruption and deletion of the *hfq *gene in the wild-type strains 2011 and 1021, respectively, both exhibiting similar growth defects as free-living bacteria. Transcriptomic profiling of 1021Δ*hfq *revealed a general down-regulation of genes of sugar transporters and some enzymes of the central carbon metabolism, whereas transcripts specifying the uptake and metabolism of nitrogen sources (mainly amino acids) were more abundant than in the wild-type strain. Proteomic analysis of the 2011-3.4 mutant independently confirmed these observations. Symbiotic tests showed that lack of Hfq led to a delayed nodulation, severely compromised bacterial competitiveness on alfalfa roots and impaired normal plant growth. Furthermore, a large proportion of nodules (55%-64%) elicited by the 1021Δ*hfq *mutant were non-fixing, with scarce content in bacteroids and signs of premature senescence of endosymbiotic bacteria. RT-PCR experiments on RNA from bacteria grown under aerobic and microoxic conditions revealed that Hfq contributes to regulation of *nifA *and *fixK1/K2*, the genes controlling nitrogen fixation, although the Hfq-mediated regulation of *fixK *is only aerobiosis dependent. Finally, we found that some of the recently identified *S. meliloti *sRNAs co-inmunoprecipitate with a FLAG-epitope tagged Hfq protein.

**Conclusions:**

Our results support that the *S. meliloti *RNA chaperone Hfq contributes to the control of central metabolic pathways in free-living bacteria and influences rhizospheric competence, survival of the microsymbiont within the nodule cells and nitrogen fixation during the symbiotic interaction with its legume host alfalfa. The identified *S. meliloti *Hfq-binding sRNAs are predicted to participate in the Hfq regulatory network.

## Background

Hfq is a ubiquitous and abundant bacterial protein which assembles into ~12 kDa ring-shaped homohexamers that resemble those formed by the Sm proteins of the eukaryotic splicing complex [1,2]. It was originally identified in the model bacterium *Escherichia coli *as a host factor essential for Q*β *RNA bacteriophage replication [3]. In uninfected bacteria Hfq retains the ability to bind many mRNAs and *trans*-acting antisense small non-coding regulatory RNAs (sRNAs), thereby influencing, directly or indirectly, on the stability and/or translation of functionally diverse RNA molecules [4-6]. This variety of interactions place Hfq at a crucial node in bacterial post-transcriptional regulatory networks underlying a wide range of cellular processes and pathways [6-8]. Consequently, mutations in the *hfq *gene were early observed to have a severe impact on bacterial physiology resulting in alterations in growth rate, cell morphology and tolerance to harsh environments [9]. In several enterobacteria and other facultative intracellular mammal pathogens these deficiencies ultimately compromise virulence traits such as motility, host invasion or growth/survival in the intracellular niche [10-16]. The virulence-related phenotypes of the *hfq *mutants have been shown to be largely dependent on the deregulation of the membrane homeostasis and RpoS- or RpoE-mediated stress response pathways, which have been reported to involve the activity of sRNAs in some of these pathogenic bacteria [15,17-19].

The α subdivision of the proteobacteria includes diverse species which share the capacity to establish a variety of long-term interactions with higher eukaryotes [20]. The pleiotropic phenotype conferred by *hfq *mutations is also common to all α-proteobacteria representatives in which the Hfq function has been genetically addressed. For example, in *Brucella *spp. the Hfq defective mutants showed osmosensitivity, reduction in the fitness of long-term cultures and impaired survival into host macrophages, further supporting the relevant role of this protein in the establishment and maintenance of chronic intracellular infections [21,22]. Besides its general contribution to stress adaptation Hfq has been also shown to influence the nitrogen fixation process in free-living (*Rhodobacter capsulatus*) and symbiotic (*Azorhizobium caulinodans *and *Rhizobium leguminosarum *bv. *viciae*) α-proteobacterial diazotrophs [23-26]. In these microorganisms Hfq acts as a positive post-transcriptional regulator of *nifA*, the gene encoding the major transcriptional activator of the genes coding for the nitrogenase complex. However, in contrast to the situation in *A. caulinodans *Hfq is not essential for the diazotrophic growth of the phototrophic purple bacterium *R. capsulatus *[24,25].

*Sinorhizobium meliloti *belongs to the group of α-proteobacterial species (collectively called rhizobia) able to engage in symbioses with legume plants. The outcome of these interactions is the formation of new specialized organs within the host, the root nodules, where bacteria undergo a process of profound morphological differentiation to their endosymbiotic form, the bacteroid. The nodules provide the microoxic environment demanded by the rhizobial nitrogenases to catalyze the reduction of the chemically inert atmospheric dinitrogen to ammonia that can be metabolized by the plant. The *S. meliloti*-*Medicago truncatula (sativa) *symbiosis is a recognized tractable model system for deciphering molecular mechanisms employed by the infective rhizobia in their transition from a free-living state in soil to their final residence within the nodule cells [27,28]. Despite the emerging role of Hfq in the establishment of successful prokaryote-eukaryote interactions, the functions of this RNA chaperone in α-proteobacteria, and in particular in the nitrogen-fixing endosymbionts, have remained largely unexplored. Nonetheless, a recent study has revealed the influence of Hfq on the stability of known *S. meliloti *sRNAs, thus anticipating the importance of this protein in sRNA-mediated regulatory pathways in this model symbiotic bacterium [29]. Here, we have determined global Hfq-dependent changes in gene expression and protein accumulation coupled with the characterization of the symbiotic behavior of *hfq *knock-out mutants to pinpoint the function of this RNA chaperone in the alfalfa symbiont *S. meliloti*. We found that loss of *hfq *alters growth and energy-producing carbon metabolic pathways in free-living bacteria, and severely compromises the nodulation competitiveness and the efficiency of the symbiosis with alfalfa. Furthermore, we provide experimental evidence of Hfq binding to some of the recently identified *S. meliloti *sRNAs [30], which predicts that these molecules could be major players in the rhizobial Hfq regulatory network.

## Results

### The *S. meliloti hfq* genomic region

**Figure 1:**
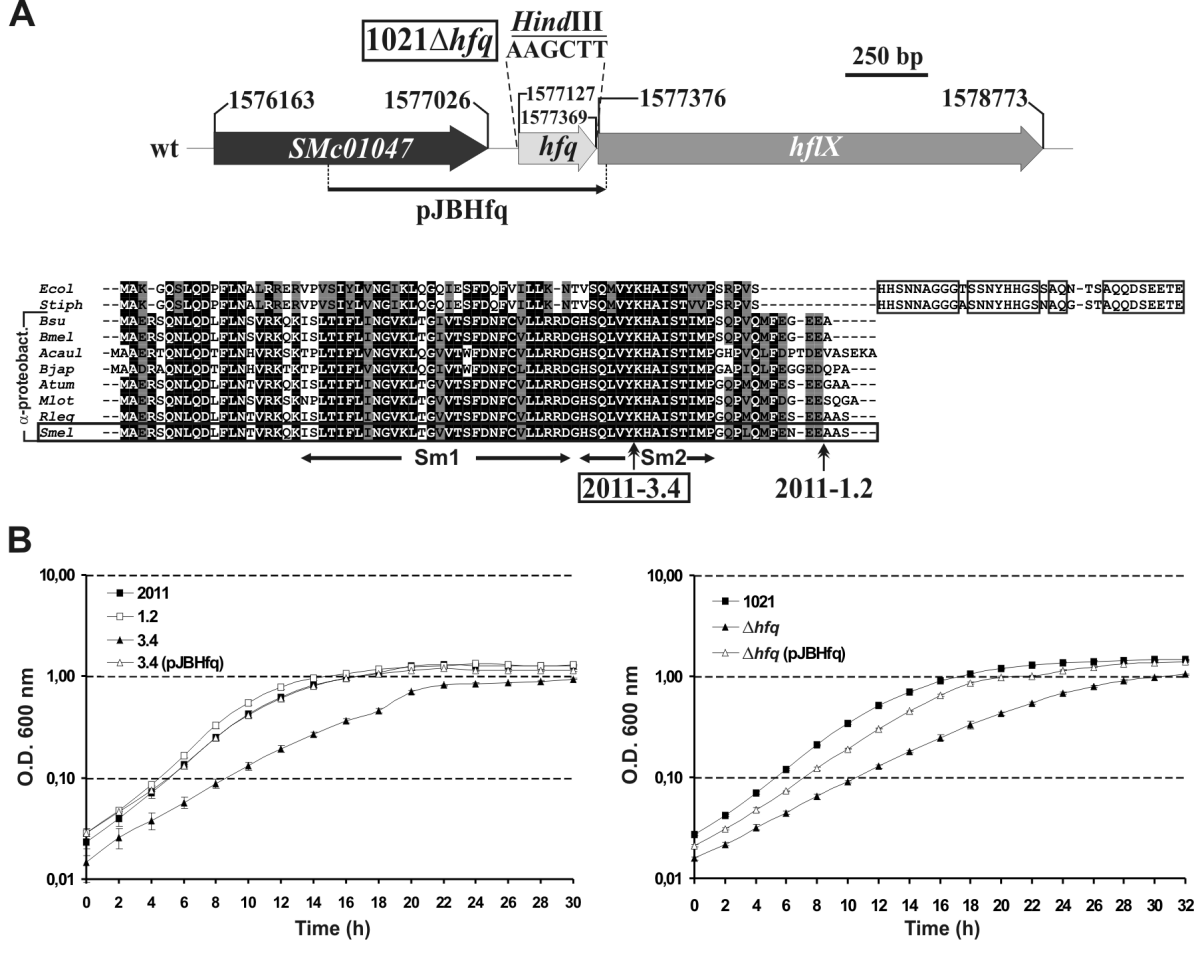
**Mutational analysis of the *S. meliloti hfq *gene**. (a) Arrangement of the genomic *hfq *region, multiple amino acid sequence alignment of Hfq proteins encoded by enterobacterial and α-proteobacterial genomes and details of the *hfq *mutants. The genetic map is drawn to scale. Numbering denotes the gene coordinates in the *S. meliloti *genome database. In the 1021Δ*hfq *mutant the full-length Hfq ORF was replaced by a *Hind*III site. The DNA fragment cloned on complementation plasmid pJBHfq is indicated. In the alignment, Hfq sequences are denoted by the species abbreviation as follows: *Ecol, E. coli; Stiph, Salmonella tiphymurium; Bsu, Brucella suis; Bmel, B. melitensis; Acaul, Azorhizobium caulinodans; Atum, Agrobacterium tumefaciens; Mlot, Mesorhizobium loti; Rleg, Rhizobium leguminosarum; Smel, S. meliloti*. Species belonging to the α-subdivision of the proteobacteria are indicated to the left. Shadowed are the amino acid residues conserved in at least 80% sequences and boxed are the conserved amino acids within the C-terminal extension of Hfq proteins encoded by enterobacteria. The two conserved Sm-like domains are indicated. Double arrowheads indicate the integration sites of pK18*mobsacB *in 2011-3.4 and 2011-1.2 derivatives. (b) Growth curves in TY broth of the *S. meliloti *wild-type strains 2011 (left panel) and 1021 (right panel) and their respective *hfq *mutant derivatives as determined by OD_600 _readings of triplicate cultures in 2 h intervals. Graphs legends: 2011, wild-type strain; 1.2, 2011-1.2 control strain; 3.4, 2011-3.4 derivative; 3.4(pJBHfq), 2011-3.4 complemented with plasmid pJBHfq; 1021, reference wild-type strain; Δ*hfq*, 1021 *hfq *deletion mutant; Δ*hfq*(pJBHfq), Δ*hfq *complemented with pJBHfq.

The *hfq *gene corresponds to ORF *SMc01048 *(formerly denoted as *nrfA*) of the *S. meliloti *genome project (http://iant.toulouse.inra.fr/bacteria/annotation/cgi/rhime.cgi) which has been annotated at bps 1577127-1577369 in the chromosome of the reference strain 1021 [31]. It is predicted to encode an 80 amino acids-long polypeptide with 72% similarity and 45% identity to the well-characterized *E. coli *Hfq protein and 77%-100% identity to its α-proteobacterial counterparts. A multiple amino acid sequence alignment revealed that the conservation between the rhizobial and enterobacterial Hfq proteins is restricted to the predicted Sm1 and Sm2 motifs likely forming the RNA-binding pocket, whereas the enterobacterial genomes encode a protein with a C-terminal extension (Fig. 1a).

The *S. meliloti hfq *gene seems to form a dicistronic operon with the downstream *hflX*-like gene coding for a putative GTP-binding protein. Upstream of *hfq *are *SMc01047 *and *trkA *coding for a D-alanine aminotransferase and a potassium transporter, respectively (Fig. 1a). Immediately upstream of *trkA *is the gene cluster specifying the nitrogen assimilation system *ntr *(*ntrB*-*ntrC*-*ntrY*-*ntrX*). This genomic arrangement is essentially conserved in all the nitrogen-fixing endosymbionts of the order *Rhizobiales*. The exception is the absence of either the *trkA *or *SMc01047 *homologs between the *ntr *operon and *hfq *in a few species (i.e. *M. loti*, *R. leguminosarum *bv. *viciae*). In contrast, the *S. meliloti hfq *upstream region totally diverges from that of its related intracellular animal pathogens (i.e. *Brucella *sp.). Enterobacterial and α-proteobacterial genomes only conserve the *hflX *gene downstream of *hfq *in this chromosomal region.

### Construction and growth characteristics of the *S. meliloti hfq* mutants

As a first approach to address the *S. meliloti *Hfq functions *in vivo *two independent *hfq *knock-out mutants were constructed in strains 2011 and 1021. These *S. meliloti *strains are derived from the same progenitor (*S. meliloti *isolate SU47) although they exhibit some phenotypic differences in growth, induction of host gene expression or response to phosphate starvation [32,33]. Nonetheless, partial sequence and restriction analyses revealed that the 1021 and 2011 *hfq *genomic regions are identical (data not shown).

A mutant (2011-3.4) and a control strain (2011-1.2) were first generated in 2011 by disruption of *hfq *with the mobilizable suicide vector pK18*mobsacB *mediated by single homologous recombination events. PCR amplification and sequence analyses of the resulting mutant alleles revealed that in 2011-3.4 pK18*mobsacB *disrupted the predicted Sm2 domain by inserting after nt 171 of the Hfq coding sequence (Fig. 1a). In 2011-1.2, plasmid integration was mapped to nt 231 of the Hfq ORF, thus affecting the translation of the non conserved last three amino acids of the protein (Fig. 1a). Both *hfq *strains formed colonies with wild-type morphology when grown in TY agar. However, the 2011-3.4 mutant exhibited a markedly slower growth than the strain 2011-1.2, which behaved as the wild-type 2011 strain on plates (not shown). When grown in TY broth with aeration no differences were observed between the wild-type 2011 strain and its derivative 2011-1.2 whereas the *hfq *insertion mutant 2011-3.4 showed a delayed lag phase and reached the stationary phase at lower optical density (Fig. 1b). This new observation further supports that the reduced growth of the 2011-3.4 strain was due to *hfq *inactivation rather than to polar effects caused by pK18*mobsacB *integration. Furthermore, the plasmid pJBHfq expressing the *hfq *gene from its own promoter fully complemented the growth phenotype of the *hfq *insertion mutant.

A second mutant was constructed in the reference strain 1021 by pK18*mobsacB*-mediated double crossing over resulting into a complete marker-free deletion of the Hfq ORF (Fig. 1a). The growth phenotype on TY agar plates previously observed in the 2011-3.4 *hfq *insertion mutant was used as a reference to discriminate between the colonies corresponding to the 1021Δ*hfq *strain and those of the wild-type revertants after the second cross over event. A Southern hybridization further confirmed the expected genomic arrangement in the mutant (not shown). In liquid TY medium the 1021Δ*hfq *strain also exhibited reduced growth rate which was complemented with plasmid pJBHfq as expected (Fig. 1b).

Therefore, 2011-3.4 and 1021Δ*hfq *mutants displayed apparent indistinguishable free-living growth defects when compared to their respective parent strains and they have been combined in this study as independent genetic tools to identify general rather than strain-specific Hfq functions in *S. meliloti*.

### Hfq-dependent alterations of the free-living *S. meliloti *transcriptome and proteome

**Figure 2:**
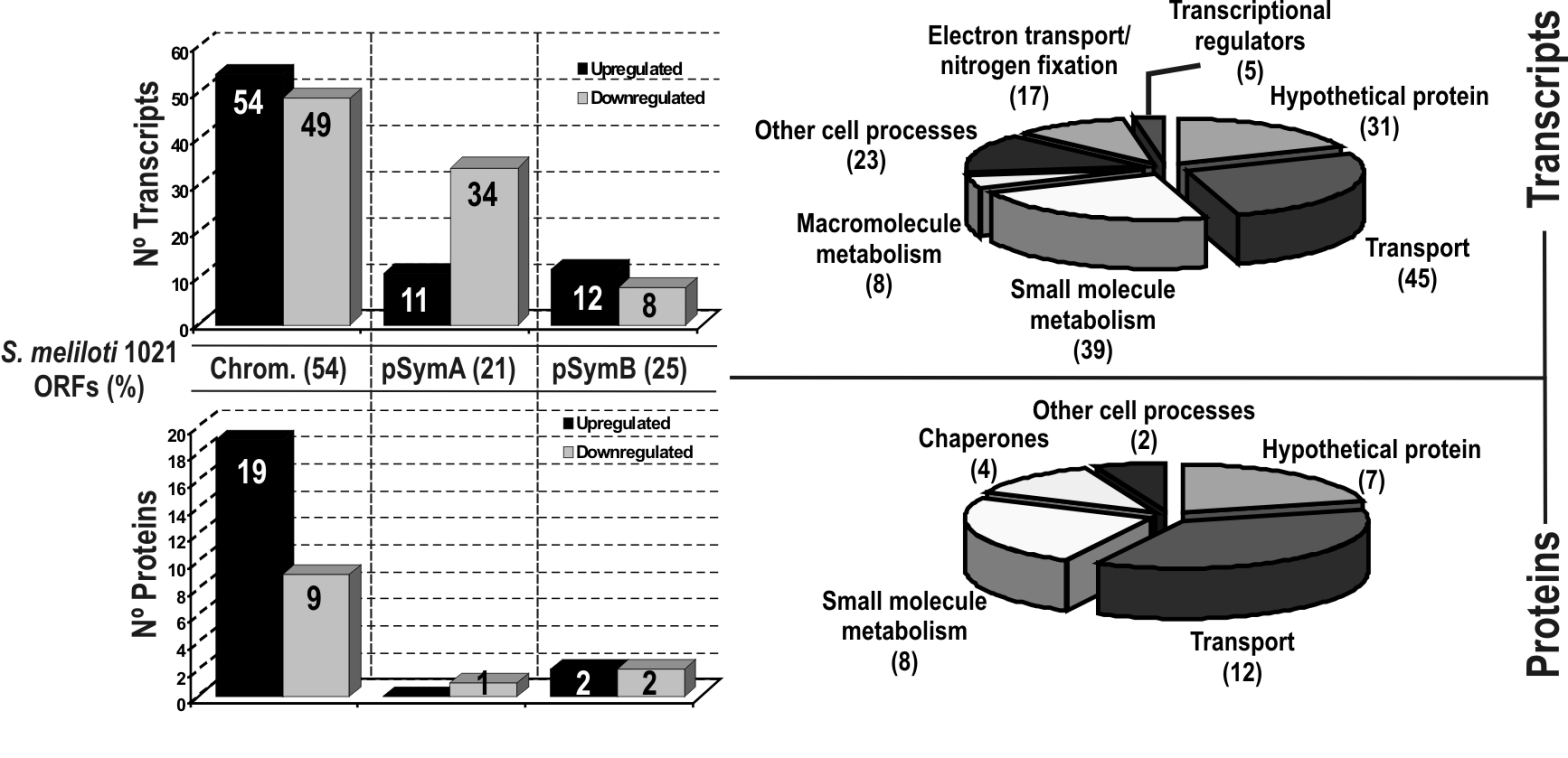
**Hfq-dependent alteration of the *S. meliloti *transcriptome and proteome**. Differentially expressed transcripts (upper graphs) and proteins (lower graphs) in the *S. meliloti hfq *knock-out mutants. Histograms show the number of differentially expressed genes and their distribution in the three *S. meliloti *replicons: chromosome (Chrom.), pSymA and pSymB. The distribution of annotated ORFs in the genome is indicated as reference. The adscription of these genes to functional categories according to the KEGG and *S. meliloti *databases is shown to the right in circle charts (see text for web pages of the referred databases). In brackets the number of genes belonging to each category.

Hfq-dependent changes in transcript abundance were first investigated by comparing the expression profiles of wild-type 1021 and 1021Δ*hfq *strains grown to lag phase (OD_600 _0.5-0.6) on whole genome Sm14kOLI microarrays (see http://www.cebitec.uni-bielefeld.de/transcriptomics/transcriptomics-facility/sm14koli.html for details on content and layout of microarrays). Hybridization signals to oligonucleotide probes corresponding to the intergenic regions were not analyzed further in this study. A total of 168 genes (2.7% of the 6206 ORFs predicted in the *S. meliloti *1021 genome) displayed at least 2-fold changes in their mRNA levels (i.e. 1 ≤ M ≤ -1) and were catalogued as differentially expressed in both strains (see additional file 1: differentially accumulated transcripts in *S. meliloti *1021 and 1021Δ*hfq *derivative strain; the microarray data described in this work have been deposited in the ArrayExpress database under accession number A-MEXP-1760). Of these, 91 were found to be down-regulated and 77 up-regulated in the 1021Δ*hfq *mutant. Replicon distribution of the 168 Hfq-dependent genes revealed that 103 (61%) were chromosomal and 65 had plasmid location; 45 (27%) in pSymA and 20 (12%) in pSymB (Fig. 2, upper charts). Taking into account the gene content of *S. meliloti *1021 with 54% genes annotated in the chromosome, 21% in pSymA and 24% located on pSymB, this distribution showed a replicon bias in Hfq activity with 1.3-fold more impact than expected on pSymA-encoded transcripts. The former observation is more evident when looking at the location of genes scored as down-regulated in the 1021Δ*hfq *mutant; as many as 34 (37%) of these 91 down-regulated genes were pSymA-borne which is almost 1.8-fold more than expected for the ORF content of this megaplasmid.

According to the *S. meliloti *1021 genome sequence annotations (http://iant.toulouse.inra.fr/bacteria/annotation/cgi/rhime.cgi)and the KEGG database (http://www.genome.jp/kegg/) 137 (82%) out of the 168 genes with altered expression in 1021Δ*hfq *could be assigned to particular functional categories, whereas 31 (18%) exhibited global or partial homology to database entries corresponding to putative genes with unknown function (Fig. 2, upper circle graph). More than half of the genes with functional homology are predicted to encode proteins and enzymes for the transport (45 genes) and metabolism (39) of small molecules, mainly sugars and amino acids, whereas the remaining 53 are related to regulation of transcription (5 genes), electron transport or nitrogen fixation (17; including the regulatory genes *fixK1/K2*) and other cell processes (31) such as macromolecule or central intermediary metabolism. Within the latter group several genes with a major role in translation and cellular RNA/protein turnover were differentially regulated in the mutant; *SMc01929 *coding for RNAseJ, *SMc03796 *encoding a putative endoribonuclease L-PSP likely involved in mRNAs cleavage, *SMa1126*, *degP4 *and *degP1 *annotated as determinants of different types of proteases, and *rplS/rpmA *both encoding ribosomal proteins. All these genes except *SMa1126 *and *degP4*, were up-regulated in the mutant.

As an independent supporting approach to investigate the Hfq function in *S. meliloti *the proteomic profiling of the wild-type strain 2011 and its *hfq *mutant derivative 2011-3.4 was also determined. Analysis of 24 Coomassie-stained 2D-gels from bacteria grown on TY medium to lag phase (OD_600 _0.5-0.8) revealed on average 293 spots of which 33 corresponded to individual polypeptides with reliable differential accumulation in the wild-type and mutant strains (see additional file 2: differentially accumulated proteins in *S. meliloti *2011 wild-type and 2011-3.4 insertion mutant derivative). Mass spectrometry (MALDI-TOF) revealed that 28 of these proteins are encoded in chromosomally located genes, 4 in pSymB and only one in the pSymA megaplasmid, thus confirming the major role of Hfq in regulating *S. meliloti *chromosomal traits (Fig. 2, lower charts). Of these 33 proteins, 21 were over-represented and 12 under-represented in the 2011-3.4 mutant strain. Classification of the differentially expressed proteins according to the *S. meliloti *1021 and KEGG databases identified three main functional categories; transport (12 proteins), small molecule metabolism (8) and chaperones and/or stress factors (4) whereas the remaining 9 were catalogued either as involved in translation (i.e. Tig trigger factor and Efp elongation factor P) or as hypothetical conserved proteins with unpredicted function (7) (Fig. 2, lower circle graph).

Comparison of the transcriptomic and proteomic profiles described in this study revealed an overlap of 9 genes identified as differentially expressed in *hfq *mutants and wild-type strains in both analyses. Their predicted encoded proteins are the periplasmic components of the ABC transporters of *myo*-inositol (IbpA), fructose (FrcB), α-glucosides (AglE), amino acids (SMc02259), leucine (LivK) and L-amino acids (AapJ and AapP) as well as two enzymes related to *myo*-inositol catabolism, IolE and IolD. Therefore, regardless the recognized phenotypic differences between the 1021 and 2011 strains both approaches support the general conclusion that Hfq has a major impact in the regulation of transport and metabolism in *S. meliloti*.

### Hfq influences central metabolic pathways in *S. meliloti*

**Figure 3:**
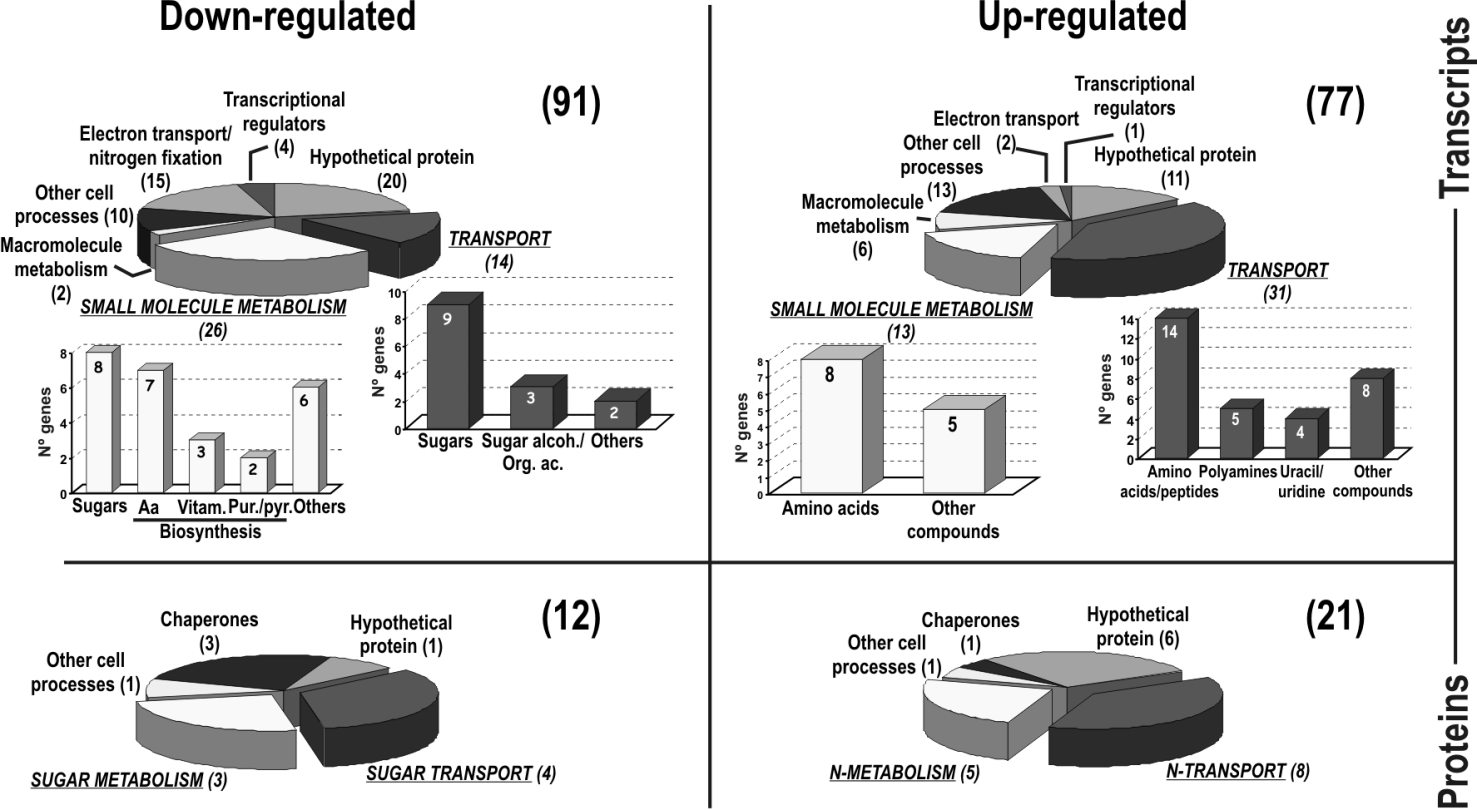
**Hfq influences central metabolic pathways in *S. meliloti***. Functional distribution of down- and up-regulated transcripts (upper graphs) and proteins (lower graphs) in the *S. meliloti hfq *mutants. In brackets is the number of genes in each category. Histograms detail the subdivision of transport and metabolic genes.

Among the 91 genes found to be down-regulated in the *hfq *deletion mutant, 40 (44%) are predicted to encode functions for the uptake (14 genes) and metabolism (26 genes) of small molecules with a major proportion related to the utilization of carbon substrates (Fig. 3, upper circle charts). Eleven of these genes form part of operons encoding the different components (i.e. the periplasmic-solute binding protein, the permease or the ATP-binding protein) of the ABC transporters for *myo*-inositol (*ibpA*, *iatA *and *iatP *genes), α-glucosides (*aglE *and *aglF*), fructose (*frcB *and *frcK*), ribose (*SMc02031*), glycerol (*SMc02514 *and *SMc02519*), and other organic acids/alcohols (*SMb20144*) [34]. An additional gene (*SMb20072*), displaying more than 32-fold reduction (M value -5.87) in transcript abundance in the *hfq *mutant has been annotated as coding for a putative *myo*-inositol-induced periplasmic solute-binding protein [34]. However, it seems to be an independent transcription unit, not clustered apparently with genes related to sugar uptake. The remaining 2 down-regulated transporter genes are likely involved in the uptake of glycine betaine (*SMc04439*) and iron (*SMc04317*). The predicted reduced efficiency in the import of primary carbon substrates by the 1021Δ*hfq *mutant was accompanied by the down-regulation of 8 genes involved in sugar catabolism: *iolC*, *iolD*, *iolE *and *iolB *integrating the operon for the utilization of *myo*-inositol, *SMc01163 *which encodes a putative glucose-fructose oxidoreductase, *SMc00982 *likely encoding a dioxygenase, and 2 putative alcohol dehydrogenase-encoding genes, *adhA1 *and *SMa1156*, predicted to be involved in fermentation of carbon substrates. Lack of Hfq also led to a reduction in the abundance of the SMa1227 transcript, which likely codes for a transcriptional regulator of the Crp superfamily, some of which have been shown to govern central carbon metabolic pathways in bacteria through cAMP binding [35]. In addition to the down-regulation of genes of energy production pathways, some transcripts encoding components of the electron transfer chain such as CycA, EtfA1 or SMa1170 (probable cytochrome c) were less abundant in the mutant. Another set of down-regulated genes in the *hfq *deletion mutant includes those involved in processes fuelled by sugar catabolism such as the biosynthesis of amino acids (*ilvC*, *SMc03211*, *SMc03253*, *pheAa*, *mtbC*, *SMc02045 *and *glyA1*), vitamins (*cobP*, *SMc04342*) and purines/pyrimidines (*purU1*, *pyrC*).

This transcriptomic profiling predicts a physiological state of bacteria demanding alternative nutrient sources to support growth and macromolecule biosynthesis in the *hfq *mutant. Indeed, our results also revealed that more than 50% of the transcripts with enhanced abundance in the mutant as compared to the wild-type strain encode proteins for the transport and catabolism of different N compounds. Up-regulated transport genes have been shown or predicted to be involved in the uptake of L-aminoacids or peptides (*aapJ*, *aapQ*, *aapP*, *oppB*, *oppC*, *SMc00140*, *SMc01597*, *SMc02259*, *SMb21572*, *SMb20605*), branched-chain aminoacids (*livH*, *livM*, *livG*, *livF*, *livK*), uracil/uridine (*SMc01823*, *SMc01824*, *SMc01825*, *SMc01827*), sugar amines (*SMb21151*) or other complex N substrates such as the polyamines spermidine and putrescine (*SMc01966*, *SMc01965*, *SMc01963*). Consequently, loss of *hfq *also resulted in the up-regulation of an important set of genes likely related to the utilization or modification of amino acids and other N compounds. The transcripts corresponding to the 3 genes specifying the glycine cleavage system, *gcvP*, *gcvH *and *gcvT *(M values 2.06, 2.02 and 3.32, respectively), and to *SMc01930 *(M value 3.26) encoding a putative methylmalonyl-CoA epimerase likely operating in the catabolism of branched-chain amino acids were particularly over-represented in the mutant.

The proteomic analysis of the other *hfq *mutant (2011-3.4) used in this study identified periplasmic solute binding proteins of ABC transporters and metabolic enzymes as the predominant sets of polypeptides which accumulation in the cell was altered by disruption of the *hfq *gene by the insertion of pK18*mobsacB *(Fig. 3, lower circle graphs). Down-regulated transport proteins are all involved in the uptake of different sugars; *myo*-inositol (IbpA), mannose/xylose/glucose (AraA), fructose (FrcB) and α-glucosides (AglE). Accordingly, several enzymes of the central carbon metabolism were also less abundant in the mutant: a putative *myo*-inositol catabolic protein (IolE), a predicted malonic semialdehyde oxidative decarboxylase (IolD) and a probable acetyl-CoA synthetase (AcsA1). Conversely, the transporters overproduced by the 2011-3.4 mutant are all related to the import of N substrates such as peptides (DppA1 and DppA2), leucine (LivK), L-amino acids (AapJ and AapP), other aminoacids (SMc02259), glycine betaine (SMc02378) or choline (ChoX). Other up-regulated proteins as a result of the *hfq *mutation include metabolic enzymes such as ornithine cyclodeaminase (Ocd), a probable arginase (ArgI1), a putative adenosylhomocysteinase (AhcY) and a phosphoenol pyruvate carboxykinase (PckA). Ocd and ArgI1 catalyze enzymatic reactions of the urea cycle whereas AhcY is involved in the metabolism of sulphur-containing aminoacids. PckA catalyzes the conversion of oxalacetate into phospho-enol pyruvate, thus initiating the gluconeogenic pathway.

In summary, transcriptomics and proteomics independently suggest that in both *S. meliloti hfq *knock-out mutants metabolism is biased towards the gluconeogenesis pathway so that growth of free-living bacteria is mainly supported by the utilization of amino acids rather than primary carbon substrates as energy sources.

### Loss of Hfq affects *S. meliloti *nodulation kinetics, competitiveness and symbiotic nitrogen fixation

**Figure 4:**
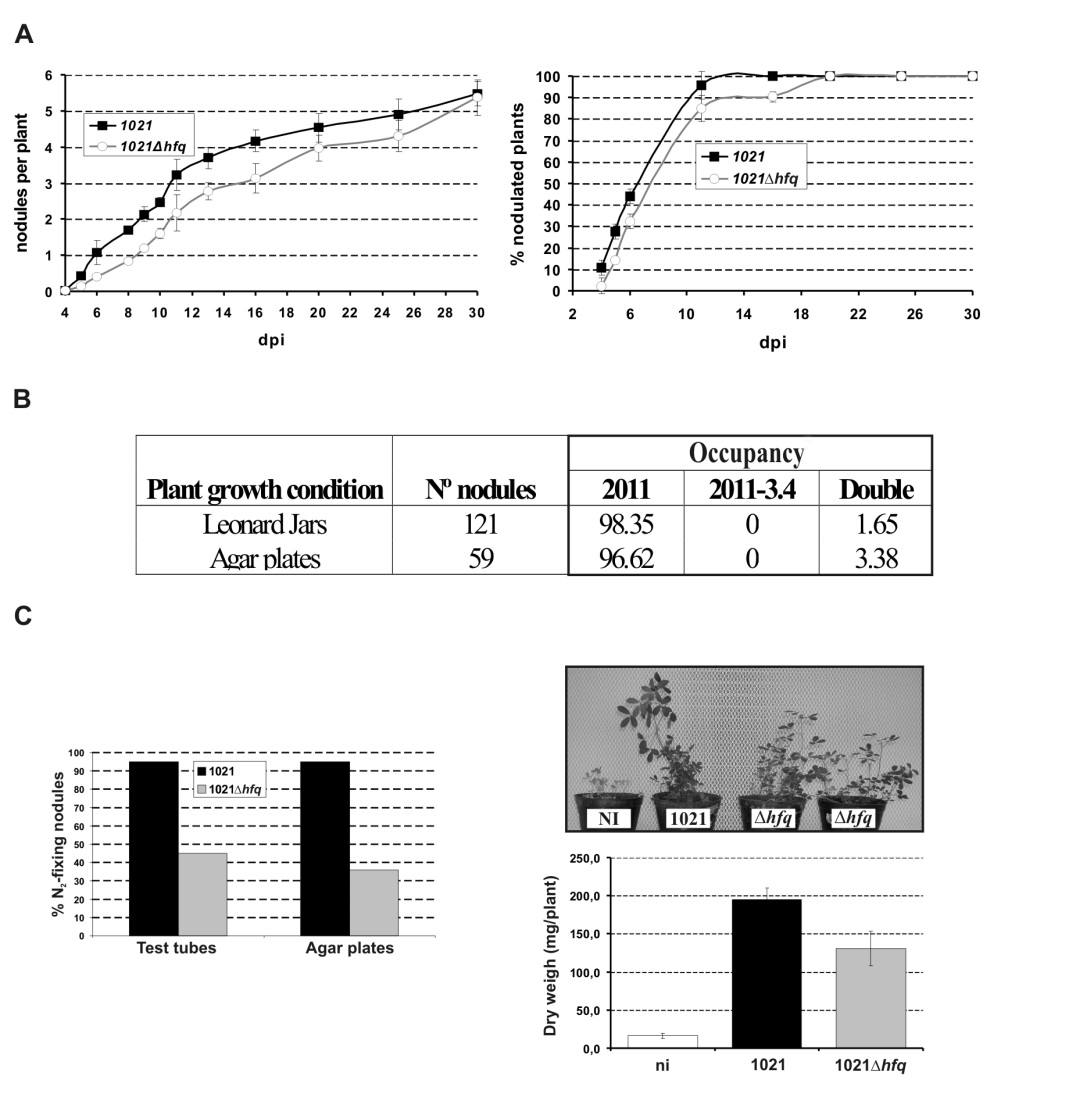
**Symbiotic phenotype of the *S. meliloti hfq *knock-out mutants**. (a) Nodule formation kinetics of the *S. meliloti *1021 wild-type strain and its mutant derivative 1021Δ*hfq *determined as the number of nodules per plant (left plot) and % nodulated plants (right plot). Each point represents the mean ± standard error of determinations in two independent sets of 24 plants grown hydroponically in test tubes. Dpi, days post inoculation. (b) Competition assays between the *S. meliloti *wild-type strain 2011 and its *hfq *insertion mutant derivative 2011-3.4. Nodule occupancy (expressed as % of invaded nodules by each strain) was determined in plants grown in either Leonard assemblies or agar plates and co-inoculated with both strains at 1:1 ratio. (c) Symbiotic efficiency of the 1021 and 1021Δ*hfq *strains. Left histogram, % nitrogen fixing nodules induced by each strain in plants grown either in test tubes (two sets of 24 plants) or agar plates (5 plates of 10 plants) 30 dpi. Right panels: growth of 1021- and 1021Δ*hfq*-inoculated plants 30 dpi in Leonard jars and dry-weigh of the same plants expressed as the mean ± standard error from measurements in 24 individual plants. Ni, not inoculated.

Nodulation and competition tests were first performed in order to evaluate the symbiotic behaviour of the *S. meliloti hfq *mutants. Sets of 24 alfalfa plants grown hydroponically in test tubes were independently inoculated with bacterial suspensions of the wild-type strains (1021 and 2011) and the knock-out *hfq *mutants (1021Δ*hfq *and 2011-3.4). The number of nodules per plant induced by each strain and the percentage of nodulated plants were recorded at daily intervals post-inoculation (dpi). No significant differences were observed in the onset of nodulation (i.e. time of appearance of the first nodule) or the average number of nodules per plant at the end of the experiment (30 dpi) when the wild-type *S. meliloti *1021 strain and the mutant 1021Δ*hfq *were compared (Fig. 4a, left plot). The *hfq *mutant was also able to nodulate 100% inoculated plants, further supporting similar nodulation efficiency of both strains (Fig. 4a, right plot). However, a discrete delay in nodulation of the mutant when compared to the wild-type nodulation kinetics was revealed by both assays. Comparison of the symbiotic behaviour of the 2011-3.4 mutant with that of its parent strain 2011 led to identical conclusions (data not shown). Together these results suggest that the loss of Hfq does not affect the ability of *S. meliloti *to elicit nodule organogenesis on alfalfa roots but it probably influences on bacterial adaptations to the plant rhizosphere.

Competition assays were then performed on alfalfa plants grown in two different solid media; Leonard assemblies and agar plates (Fig. 4b). Taking advantage of the tagging of the 2011-3.4 mutant with the Km resistance marker of pK18*mobsacB *co-inoculation suspensions were prepared in this case by mixing *S. meliloti *2011 and 2011-3.4 suspensions at 1:1 ratio. Thirty dpi nodules were able to fix nitrogen as revealed by their pink colour because of the presence of leghemoglobin. A total of 180 nodules (121 from plants in Leonard jars and 59 from plants on plates) were excised from roots, crushed and simultaneously plated on TY and TY-Km to identify nodule-occupying bacteria. Only 2.5% of the nodules analyzed (mean of the two experiments) were found to contain the mutant 2011-3.4 (Fig. 4b). Nonetheless, wild-type bacteria were also found within these nodules and therefore the former percentage represents double occupancy. The remaining nodules (97.5% on average) were exclusively occupied by the wild-type 2011 strain. These findings revealed that loss of Hfq has a major impact on nodulation competitiveness of *S. meliloti *on alfalfa roots.

Major differences in the symbiotic behaviour of the 1021 wild-type strain and the 1021Δ*hfq *mutant were also observed when looking at the final number of nitrogen-fixing nodules (i.e. pink nodules expressing the plant leghemoglobin) induced by each strain when inoculated independently on alfalfa plants. This parameter was determined in plants grown either in test tubes or agar plates. At the end of the experiment (30 dpi) 95% nodules elicited by the wild-type strain were pink as indicative of active nitrogen-fixation, whatever the plant growth conditions, whereas 55% (test tubes)-64% (agar plates) nodules induced by the 1021Δ*hfq *mutant remained white (Fig. 4c, left graph). Furthermore, the first wild-type pink nodule appeared on average 13 dpi. In contrast, this time was estimated to be 18 dpi in plants inoculated with the 1021Δ*hfq *strain. Finally, growth of alfalfa plants inoculated with *S. meliloti *1021 and 1021Δ*hfq *strains were also compared in experiments performed on Leonard jars during 30 days (Fig. 4c, right panels). Plants inoculated with the *hfq *mutant exhibited leaves with pale green colour and reached roughly half of the height of the 1021-inoculated plants. Dry weight determinations of individual plants confirmed this perception; the average weight of plants inoculated with the 1021Δ*hfq *strain was hardly 64% of that of wild-type-inoculated plants. These results indicate that Hfq also influenced late symbiotic stages and is required for the establishment of an efficient nitrogen-fixing symbiosis.

### 1021Δ*hfq*-induced nodules are almost devoid of nitrogen-fixing bacteroids

**Figure 5:**
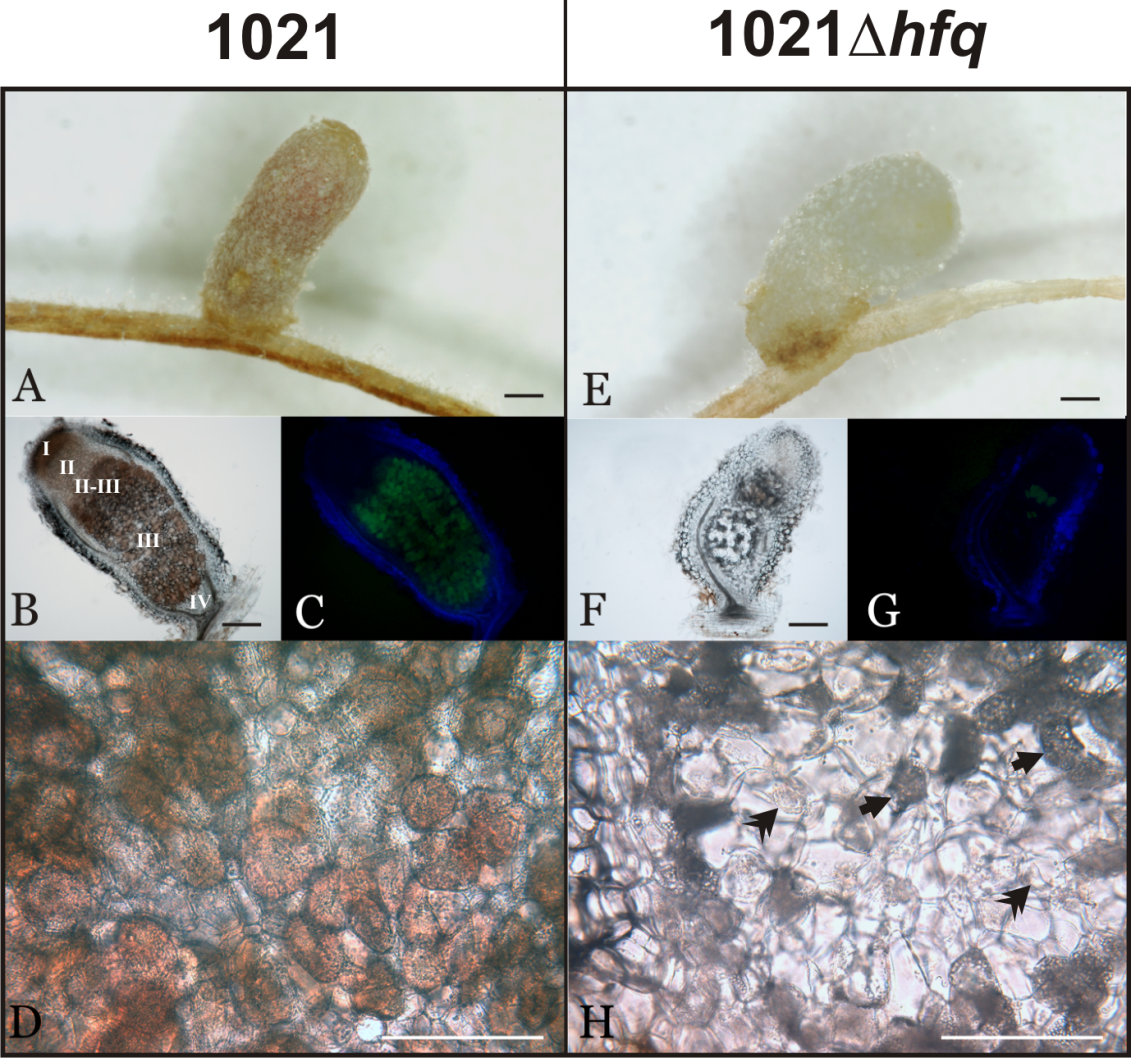
**The 1021Δ*hfq *mutant is impaired in the survival within the nodule cells**. Representative enlarged images of nodules induced in alfalfa plants by the 1021 (a) and 1021Δ*hfq *(e) strains. Bright-field microscopy of longitudinal sections of the same nodules (b and f); the zones characterizing the histology of nitrogen-fixing indeterminate nodules are indicated in (b). Merged images of the same nodule sections observed with green and blue filters (520 nm and 470 nm, respectively) (c and g). Magnification of the images of central nodule tissues (d and h); 1021Δ*hfq*-induced nodules are scarcely invaded by bacteria and show signs of premature senescence: degradation of leghemoglobin (arrows) and cell debris (double arrowheads). Scale bars, 250 μm.

To analyse in more detail the endosymbiotic phenotype associated to an *hfq *mutation we performed optical microscopy on nodules induced by the 1021 wild-type strain and its 1021Δ*hfq *mutant derivative (Fig. 5). Wild-type nodules were elongated and pink coloured as indicator of active symbiotic nitrogen fixation (Fig. 5a). Bright-field microscopy of longitudinal sections of these nodules revealed the successive zones characterizing the histology of indeterminate nodules: the apical meristem or zone I, the infection zone II; the bacteroids-infected zones II-III and III; and the proximal senescence zone IV containing plant cells appearing empty under the light microscope (Fig. 5b) [36]. Merged images of the same nodule section observed under green and blue filters (520 nm and 470 nm, respectively), confirmed the uniform colonization of central nodule tissues by differentiated green autofluorescent bacteroids (Fig. 5c). A magnification of a section of the nitrogen-fixation zone III further showed evident signs of active leghemoglobin expression in the majority of plant cells which were fully and homogeneously invaded by bacteroids that are visualized as little vesicles (Fig. 5d).

A large proportion of 1021Δ*hfq*-induced nodules were white and less elongated than those induced by the wild-type strain, thus revealing symbiotic deficiencies (Fig. 5e). The remaining nodules appeared pink and exhibited wild-type histology (not shown). Light microscopic observation of longitudinal sections of the Fix^-^-looking nodules revealed that the bacteroid-infected tissues were restricted to the interzone II-III which even showed much less autofluorescence than in wild-type nodules when observed under 520 nm light (Fig. 5f and 5g). The underlaying zone, extending to the base of the nodule, did not look as a typical nitrogen-fixation zone III but instead it resembled the senescence tissues (zone IV) of wild-type nodules. A detail of this zone (Fig. 5h) further evidenced the histological reminiscences of zone IV where a major proportion of plant cells were devoid of differentiated bacteria and started to collapse as revealed by the appearance of some cell debris [37]. The few plant cells housing bacteroids were not pink as in the wild-type nodules, but rather they appeared dark, probably because of leghemoglobin degradation concomitant to bacterial death. We interpret this histology as the 1021Δ*hfq *mutant retained some capacity to infect the host and to differentiate into bacteroids but it was compromised in the survival as endosymbiotic bacteria within the nodule cells. This deficiency is the major determinant of the Fix^- ^phenotype observed in these nodules.

### Hfq contributes to the regulation of *nifA *and *fixK1/K2*

**Figure 6:**
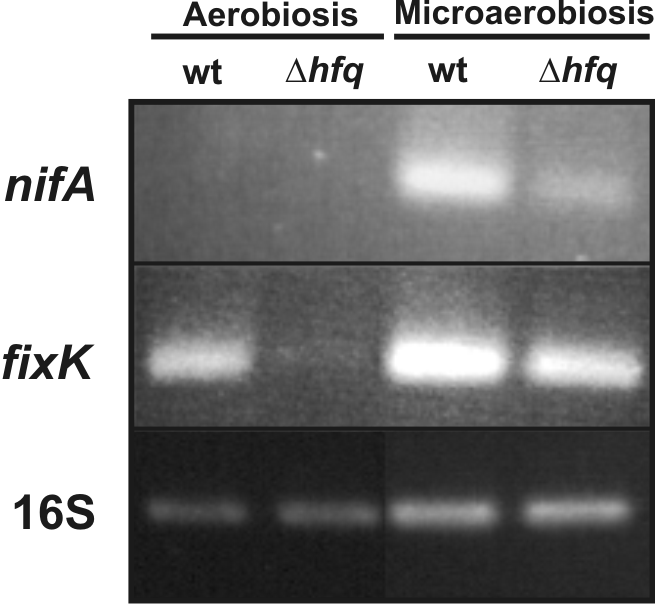
**Hfq contributes to the regulation of *nifA *and *fixK *expression**. RT-PCR analysis on RNA extracted from the wild-type strain 1021 (lanes 1 and 3) and the *hfq *mutant (lanes 2 and 4) before (lanes 1 and 2) and after (lanes 3 and 4) culture incubation for 4 h in microaerobiosis (2% O_2_). 16S was amplified as constitutive control of expression. Mock-treated (no RT) RNA samples were also PCR amplified with the same primer combinations to check for absence of DNA contamination (not shown).

An important set of pSymA-encoded transcripts with a decreased accumulation in the 1021Δ*hfq *mutant included those corresponding to the two copies of the *fixK *regulator (*fixK1 *and *fixK2*) and their downstream-dependent genes coding for the components of the respiratory chain associated to the nitrogenase complex (*fixN1*, *fixQ1*, *fixP1*, *fixG*, *fixQ2*, and the two copies of *fixM*) (Fig. 3, upper circle graph). This was a surprising finding since it is well documented that transcription of nitrogen fixation genes (*fix/nif*) is oxygen-regulated in legume nodules and only induced under microoxic conditions in free-living bacteria [38]. Nonetheless, it has been also reported that a moderate decrease of the ambient oxygen concentration (to 5%) in the gas phase over a culture is sufficient to trigger ATP-dependent autophosphorylation of the deoxygenated FixL hemoprotein in the FixLJ-FixK phosphorelay cascade [39]. In *S. meliloti *phosphorylated FixJ not only activates transcription of the *fixK1/K2 *regulatory genes but also of *nifA*, the transcriptional activator of the *nif *genes specifying the nitrogenase complex. Expression of *nifA *has been shown to demand more stringent microaerobic conditions [38]. Therefore, down-regulation of the *fix *genes in the *hfq *mutant can be only explained if our culture conditions (15-ml test tubes) enabled some level of expression of *fixK1*/*fixK2 *in the wild-type 1021 strain and the accumulation of the corresponding transcripts is influenced by the lack of Hfq. Indeed, β-galactosidase assays in the wild-type 1021 strain carrying a *fixK::lacZ *transcriptional fusion demonstrated a 4-fold induction of *fixK *transcription in our culture conditions compared to better aerated cultures (i.e. 20-ml cultures in 100-ml Erlenmeyer flasks). Similar experiments with a *nifA::lacZ *transcriptional fusion revealed no signs of transcription of *nifA *whatever the aeration of the culture (not shown). These findings and the fact that *nifA *expression had been also shown to be influenced by Hfq in other α-proteobacterial diazotrophs [23-26] prompted us to further investigate the effects of Hfq on both *nifA *and *fixK *expression in more stringent microaerobic conditions by RT-PCR (Fig. 6). Confirming the results of microarray experiments FixK transcripts were readily detected in RNA from wild-type bacteria grown under assumed aerobiosis (Fig. 6; line 1), whereas the 1021Δ*hfq *failed to accumulate these transcripts in these culture conditions (Fig. 6; line 2). As expected, after 4 hours incubation in a microoxic atmosphere (2% O_2_) wild-type *fixK *expression was clearly induced as compared to aerobiosis (Fig. 6; compare lines 1 and 3). Strikingly, similar amounts of the FixK transcript were detected in the RNA from the *hfq *mutant extracted after the same treatment (Fig. 6; line 4). In contrast, *nifA *expression was only detected after bacterial incubation in microaerobiosis (Fig 6; line 3), further confirming that transcription of this gene demands lower O_2 _concentrations than *fixK*. A significant reduced amount of NifA amplification product was detected in the 1021Δ*hfq *mutant RNA, although this was still visible in ethidium bromide stained gels (Fig. 6; line 4). Together, these results indicate that full expression of *fixK *and *nifA *requires Hfq. Nonetheless, Hfq-mediated regulation of *fixK *does not operate under *in vitro *microoxic conditions and, therefore it could not be relevant to symbiosis.

### Some *S. meliloti *sRNAs bind Hfq

**Figure 7:**
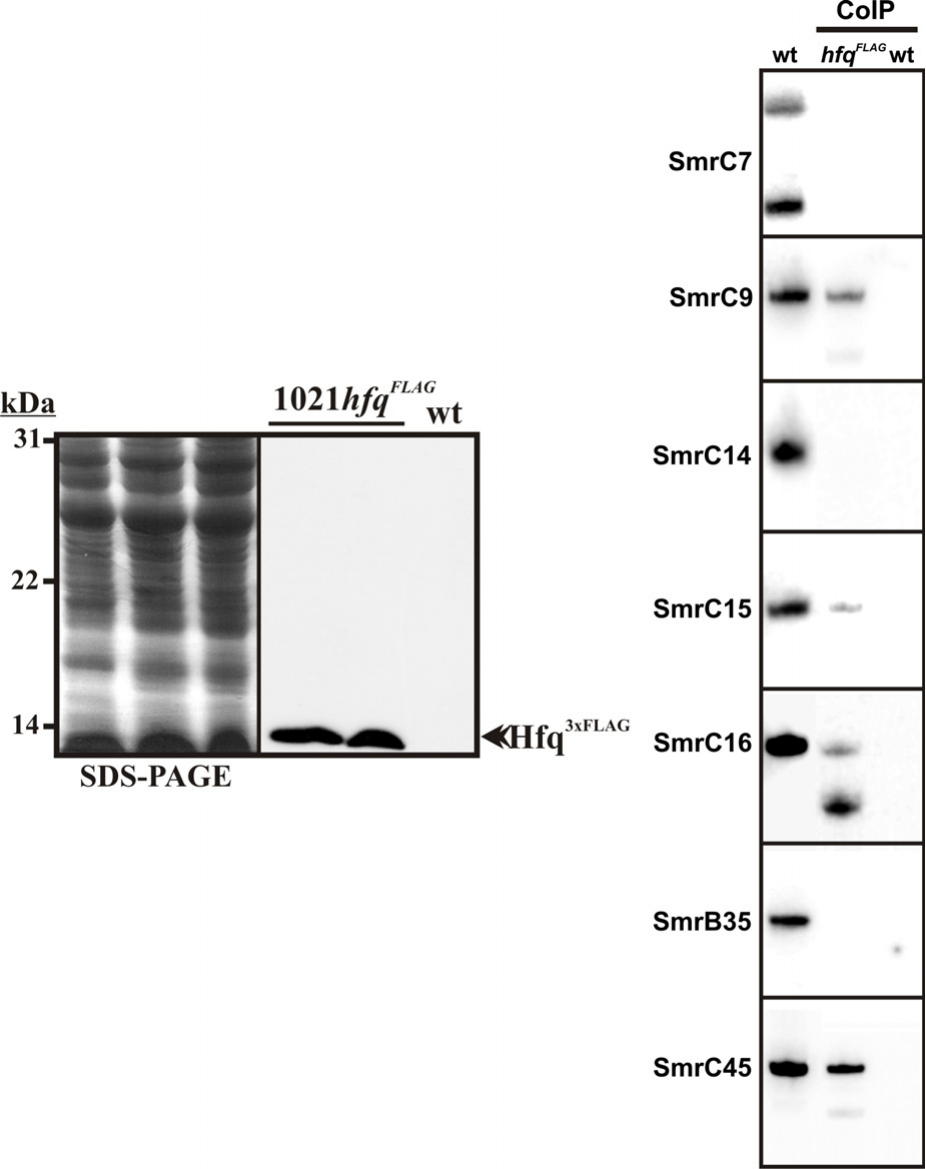
**Binding of *S. meliloti *sRNAs to a FLAG-epitope tagged Hfq protein**. Western-blot showing the specific recognition of the chromosomally encoded 3 × FLAG tagged Hfq protein by ANTI-FLAG M2^® ^monoclonal antibodies in total protein extracts of two independent 1021*hfq*^FLAG ^strains (i.e. two different clones arising from the second cross-over event) (left panel); and Northern analysis of CoIP RNA from the 1021*hfq*^FLAG ^and wild-type strains for the detection of the Smr sRNAs (right panel). Lane 1 shows the expression pattern of the corresponding sRNAs in the wild-type strain.

Mechanisms underlying Hfq-dependent post-transcriptional regulation of gene expression could involve interaction of the protein with either mRNA or sRNA molecules. We have recently reported on the computational prediction and experimental validation of seven *S. meliloti *sRNAs, denoted as Smr RNAs, exhibiting differential expression patterns potentially relevant to symbiosis [30]. To test which of these Smr transcripts are Hfq targets we have used RNA co-inmunoprecipitation (CoIP) with a chromosomally-encoded FLAG epitope-tagged Hfq protein specifically recognized by monoclonal anti-FLAG antibodies in cell extracts of a *S. meliloti hfq*^FLAG ^strain (Fig. 7, left panel). This modification did not alter the growth phenotype of the wild-type strain (not shown), thus suggesting that the tagged variant of the *S. meliloti *Hfq protein is uncompromised in its ability to bind RNA, as reported in other bacterial species [40]. CoIP RNAs were subjected to Northern analysis with oligonucleotide probes for the Smr RNAs [30]. For each sRNA, Hfq binding was assessed at the growth phase in TY broth where the sRNA was previously shown to be most abundant; log phase for transcripts SmrC7, SmrC9, SmrC14, SmrC16, SmrB35 and SmrC45 and stationary phase for SmrC15. As a control of binding specificity, identical analyses were performed in extracts from the wild-type strain 1021 which does not express any polypeptide recognized by the anti-FLAG antibodies (Fig. 7, left panel). As expected, no hybridization signal was detected for any of the tested sRNAs in CoIP samples from this control strain (Fig. 7, right panel). In contrast, hybridization bands corresponding to SmrC9, SmrC15, SmrC16 and SmrC45 full-length transcripts were readily detected in CoIP RNA from the *S. meliloti hfq*^FLAG ^strain and thus, they were concluded to specifically bind to the epitope-tagged Hfq protein (Fig. 7, right panel). Comparison of Smr transcripts abundance in the CoIP samples and their expression levels in *S. meliloti *likely revealed different binding efficiencies of these sRNAs to Hfq. Therefore, the lack of hybridization signals to SmrC7, SmrC14 and SmrB35 probes could be interpreted as either absence in the CoIP RNA or very low binding affinity to Hfq which renders these sRNAs under the detection level of the Northern hybridization. Additional bands of different intensity, not detected in the *S. meliloti *total RNA, corresponding to RNA species smaller than the full-length transcripts were also visible when CoIP RNA was hybridized to SmrC9, SmrC16 and SmrC45 probes. A recent report addressing the stability of the seemingly homologous SmrC15 and SmrC16 sRNAs in a *S. meliloti *2011 Δ*hfq *mutant suggested that Hfq protects both full-length transcripts from degradation and stabilises degradation products corresponding specifically to the 3'-half of SmrC16 [29]. Our results corroborate that both, SmrC15 and SmrC16 sRNAs do bind Hfq and also suggest that the major band detected by the SmrC16 probe could correspond to a degradation product of this transcript interacting with a particular high efficiency with the protein. Nonetheless, the identity of this SmrC16-derived product remains controversial since the probe used in our study hybridizes to the 5'-half rather than to the 3'-end of the full-length transcript. Thus, further verifications should be carried out to elucidate this apparent contradiction. Similarly, the additional faint hybridization bands detected with SmrC9 and SmrC45 probes could be interpreted as corresponding to degradation products of these sRNAs retaining a less efficient binding capacity to Hfq than the full-length transcripts.

## Discussion

**Figure 8:**
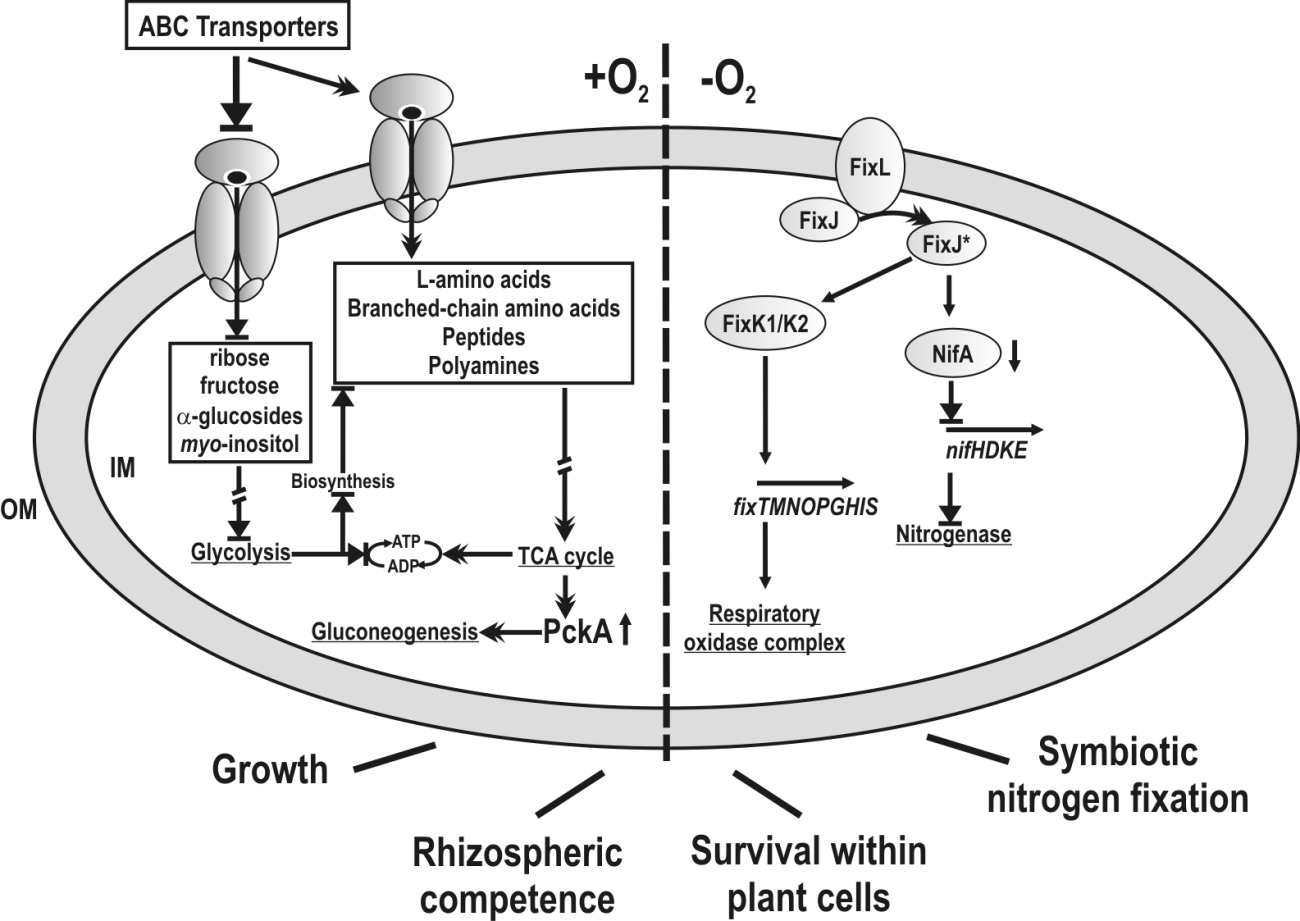
**Summary of pathways and phenotypes linked to an *hfq *mutation in *S. meliloti***. Double arrowheads denote favoured pathways and blocked arrows unfavoured pathways in the absence of Hfq. +O_2_, aerobic conditions; -O_2_, microaerobic conditions.

There is increasing evidence that the ubiquitous RNA chaperone Hfq acts as a global post-transcriptional regulator controlling gene networks underlying key steps in the interactions of pathogenic bacteria with their eukaryotic hosts [41]. However, its role in beneficial host-microbe interactions had not been investigated in detail. Here, we have genetically addressed the function of Hfq in the nitrogen-fixing endosymbiont *S. meliloti*, both as free-living bacterium and during the symbiotic interaction with its legume host alfalfa. As summarized in the model shown in Fig. 8, our results suggest the involvement of Hfq in bacterial pathways affecting central metabolism, rhizospheric competence, survival within the nodule cells and symbiotic nitrogen fixation. Hfq had been previously reported to have a role in the regulation of nitrogen fixation genes in several α-proteobacterial diazotrophs [23-26]. Therefore, the present study extends the role of Hfq in beneficial nitrogen-fixing bacteria to other processes related to the interaction with the plant host, further supporting the predicted universal role of Hfq in the establishment and maintenance of chronic intracellular residences regardless the outcome of these infections. Furthermore, we provide the first experimental evidence of *S. meliloti *sRNAs-binding Hfq, thus anticipating the involvement of these molecules at different levels in the complex *S. meliloti *Hfq regulatory network.

### Hfq influences growth and central carbon metabolism in *S. meliloti*

Hfq loss-of-function affected the free-living growth of *S. meliloti*, thus confirming the predicted pleiotropy of this mutation in bacteria. To investigate the molecular basis of this growth deficiency we combined transcriptomic and proteomic profiling of two independent *S. meliloti hfq *mutants (1021Δ*hfq *and 2011-3.4) exhibiting similar free-living growth defects. These experiments identified 168 transcripts and 33 polypeptides displaying reliable differential accumulation in the respective mutant and wild-type strains, with 9 genes common to both sets. The differences between the wild-type 2011 and 1021 strains could partially explain the limited overlap between proteins and transcripts regulated by Hfq in both genetic backgrounds. However, this has been also observed in *Salmonella *and more likely reflects the differential global effects of this protein on transcription, transcript stability and translation [42]. Nonetheless, both analyses converged in the identification of genes coding for periplasmic solute binding proteins of ABC transporters and metabolic enzymes as the dominant functional categories influenced by an Hfq mutation. The extensive role of Hfq in the regulation of nutrient uptake and central metabolism has been also highlighted by global transcriptome/proteome analyses of other *hfq *mutants such as those of *E. coli*, *Salmonella tiphymurium*, *Pseudomonas aeruginosa *or *Yersinia pestis *[15,43-45]. Furthermore, in *Salmonella *and *E. coli *the massive regulation of genes encoding periplasmic substrate-binding proteins of ABC uptake systems for amino acids and peptides involves the Hfq-dependent GcvB sRNA [46]. GcvB homologs of distantly related bacteria conserve a G/U-rich stretch that binds to extended complementary C/A-rich regions, which may serve as translational enhancer elements, in the mRNA targets [46]. The apparent widespread distribution of GcvB RNAs in bacteria suggests that a similar regulatory mechanism for ABC transporters could also exist in *S. meliloti*. Loss of Hfq also resulted in the general down-regulation of genes of the main central energy production pathways based on sugar uptake and catabolism as well as of some other genes predicted to participate in the biosynthesis of macromolecule building blocks such as amino acids or nucleosides. This seems to impose a metabolic shift favouring TCA and gluconeogenesis which are supported by the up-regulation of the amino acid supply and nitrogen metabolism (Fig. 8). Furthermore, some genes encoding components of the electron transfer chains were also down-regulated in the mutant, which predicts the reduction of the proton motive force across the cytoplasmic membrane. We conclude that this metabolic rearrangement could explain the growth phenotype of the *S. meliloti hfq *knock-out mutants.

### Lack of Hfq affects different stages of the *S. meliloti*-alfalfa symbiosis

Early events of the symbiotic interaction of rhizobia with their legume hosts involve active colonization of the plant rhizosphere and the subsequent response to specific root-exuded compounds (i.e flavonoids) to trigger Nod factor signalling pathways leading to nodule organogenesis [27,28,47]. The rhizosphere is a complex environment providing bacteria with a wide range of carbon and nitrogen compounds. Therefore, the ecological success of the legume symbionts demands high metabolic plasticity, which in *S. meliloti *is guaranteed by the large sets of genes encoding ABC transporters and metabolic enzymes [31]. It is well documented that metabolic traits related to carbon supply and catabolism are important for *S. meliloti *to successfully compete for nodulation in the rhizosphere [48]. We have shown that the *S. meliloti hfq *mutants, when independently inoculated, are able to nodulate alfalfa roots at similar rates than the wild-type strains; although a slight delay in nodulation was observed. These results evidence that the *hfq *mutation did not compromise the perception and production of the specific symbiotic signals (i.e. flavonoids and Nod factors, respectively) that trigger nodule organogenesis but suggest that bacterial adaptation in the rhizosphere was affected. Indeed, in the presence of the wild-type strain an *hfq *knock-out mutant was unable to elicit nodules, further supporting that the metabolic alterations linked to the loss of Hfq represent a major disadvantage for the competitive colonization of the alfalfa rhizosphere.

Although the *S. meliloti hfq *mutants were able to induce nodules on alfalfa roots (Nod^+ ^phenotype) we noticed that a large proportion of these nodules looked non-fixing. Furthermore, we also observed a significant delay in the onset of symbiotic nitrogen-fixation (i.e. expression of the leghemoglobin) in the remaining mutant-induced nodules (36%-45%) as compared to wild-type kinetics. As expected, these symbiotic deficiencies negatively affected the outcome of symbiosis (i.e. plant growth). Together, these findings indicate an influence of Hfq in intermediate and/or late symbiotic stages. A closer examination of the white 1021Δ*hfq*-induced nodules revealed scarce presence of leghemoglobin and a small proportion of plant cells containing nitrogen-fixing endosymbiotic bacteria. Furthermore, signs of premature bacteroid senescence were observed in these nodules. These results suggest that loss of Hfq affects the ability of *S. meliloti *to survive within the intracellular environment of the host. This phenotype has been reported as a common feature of *hfq *mutants of phylogenetically distant pathogenic bacteria [10-12,15,21,22].

Legumes provide invading bacteria with defined and dominant energy sources (i.e. dicarboxylic acids for bacteroids) other than the carbon substrates used for free-living growth in the rhizosphere [47]. Therefore, although the alteration of central metabolic pathways could contribute to different extent to the colonization of developing nodules, they provide only a partial explanation for the *hfq *endosymbiotic phenotype. Besides nutrient compounds, invading bacteria has to perceive and respond to a variety of plant signals to successfully colonize legume nodules [27,28], these include; reactive oxygen species released by the host upon infection [49], peptides likely transported into bacterial cells by the product of the *bacA *gene to launch bacteroid differentiation [50,51], the low pH of intracellular compartments [52] or the microoxic environment demanded by the nitrogenase complex to fix atmospheric nitrogen [38]. Our proteomic analysis identified GroEL2, GroEL3, GrpE and IbpA chaperons as deregulated in the 2011-3.4 *hfq *mutant. Four *groESL *operons and an additional *groEL *gene are present in the *S. meliloti *genome, being the *groEL1 *required for nodulation and nitrogen-fixation [53,54]. Thus, it can be speculated that Hfq-dependent chaperones could help also infective rhizobia to cope with the prolonged stress within the plant host. On the other hand, the transcriptomic profiling revealed that the accumulation of FixK1/FixK2 transcripts is Hfq-dependent. RT-PCR experiments on RNA obtained from cells subjected to more stringent microaerophilic conditions revealed that Hfq-mediated regulation of *fixK *operates in our assumed aerobic conditions but not in microaerobiosis. In *S. meliloti fixK *expression is also subjected to indirect autoregulation through the inhibition of the FixL sensor kinase by the FixT protein [55,56]. Therefore, our findings add another level of complexity to the FixK-dependent regulatory circuit whose biological significance remains to be elucidated. The same RT-PCR experiments showed that Hfq also contributes to the positive regulation of *nifA*, although transcripts of this gene were still detected in the mutant. Down-regulation of *nifA *would impact on nitrogenase synthesis, thus explaining the Hfq effects on the onset and probably the efficiency of nitrogen fixation itself in 36%-45% nodules that supported growth and development of the 1021Δ*hfq*-inoculated plants in our assays. In addition, it has been recently reported that the effects of a mutation in the *nifA *gene extends beyond the regulation of the nitrogen fixation genes to influence other cellular processes such as motility or synthesis of extracellular proteins [57]. Therefore, the down-regulation of this gene provides further explanation for the symbiotic phenotype of the *hfq *mutant. It has been recently reported that the Hfq-mediated post-transcriptional regulation of *nifA *in *R. leguminosarum *bv. *viciae *involves the cleavage of NifA mRNA in its 5' region by RNAseE, thereby making the Shine-Dalgarno sequence accessible for the ribosomes [26]. Given the synteny of the *nifA *genomic region in *S. meliloti *and *R. leguminosarum *it is tempting to speculate on a similar mechanism controlling NifA translation in the alfalfa endosymbiont.

Detailed genome-wide identification of Hfq-dependent symbiotic genes *in planta *is a technical difficult task that can be approached by mimicking specific symbiotic conditions in bacterial cultures. Therefore, our study is definitely worth extending to all abiotic and biotic stresses impacting the *S. meliloti *symbiotic lifestyle. Nonetheless, the similarities among *hfq*-related phenotypes in phylogenetically distant bacterial species anticipate a conservation of major Hfq downstream target genes governing common adaptive responses of bacteria for the interaction with and the invasion of their eukaryotic hosts.

### Some *S. meliloti *sRNAs are Hfq targets

*Trans*-acting antisense regulatory sRNAs are major components of Hfq-dependent regulatory networks helping bacteria to deal with external stimuli [5,8,58,59]. Cellular processes controlled by Hfq-binding sRNAs include quorum sensing, transport and metabolism, synthesis of virulence factors, sensitivity to antimicrobial peptides or general adaptation to a variety of abiotic stresses including low pH or oxidative stress [41]. Therefore, many of the recently identified *S. meliloti *sRNAs are predicted to fulfil similar functions in an Hfq-dependent manner [30,60,61]. We used a genetically modified *S. meliloti *1021 strain expressing a chromosomally-encoded FLAG-epitope tagged Hfq protein to search for Hfq targets among the seven differentially expressed sRNAs identified and mapped in our previous work [30]. This is a generic strategy that has been shown to retrieve high amounts of Hfq-binding RNAs with high specificity [40,59,62]. Our CoIP experiments identified 4 out of the 7 sRNA transcripts as specific targets of Hfq: SmrC9, SmrC15, SmrC16 and SmrC45. Accordingly, the conserved secondary structure of these sRNAs, as inferred from co-variance models, revealed several single stranded AU-rich stretches (del Val and Jiménez-Zurdo, unpublished) which are predicted to interact with Hfq [6]. *S. meliloti *encodes an Hfq protein conserving the RNA binding core but lacking the C-terminal extension of γ- and β-proteobacterial Hfqs. In *E. coli *this C-terminal domain is dispensable for sRNA binding but required for auto- and riboregulation [63]. Therefore, the mechanistic and biological implications of sRNAs binding to a naturally-occurring truncated variant of Hfq remain as open questions.

## Conclusions

The *S. meliloti *RNA chaperone Hfq is a pleiotropic regulator influencing central metabolic pathways in free-living bacteria and several aspects of the symbiosis with its legume host alfalfa: nodulation competitiveness, survival of endosymbiotic bacteria within the nodule cells and expression of the key regulators of nitrogen-fixation. The identified Hfq-dependent phenotypes, mRNAs and sRNAs in a beneficial plant-interacting rhizobacteria such as *S. meliloti *constitute a new baseline to further investigate the Hfq-mediated pathways controlling common strategies of phylogenetically distant bacteria to colonize, infect and survive within their eukaryotic host cells.

## Methods

### Bacterial strains, plasmids, media and growth conditions

Bacterial strains and plasmids used in this study along with their relevant characteristics are listed in Table 1. *S. meliloti *wild-type and *hfq *mutant derivative strains were routinely grown in complex tryptone-yeast TY [64] or defined MM media [65] at 30°C and *E. coli *strains in Luria-Bertani (LB) medium at 37°C. For microaerobic growth bacteria were initially grown in 25 ml of TY medium in aerated shaken flasks to O.D_600 nm _0.5. Cultures were then flushed with a 2% oxygen-98% argon gas mixture during 10 min and incubated for a further 4 h. Antibiotics were added to the media when required at the following final concentrations: streptomycin (Sm), 250 μg/ml; ampicillin (Ap), 200 μg/ml; tetracycline (Tc), 10 μg/ml; and kanamycin (Km), 50 μg/ml for *E. coli *and 180 μg/ml for rhizobia.

**Table 1:** Bacterial strains and plasmids.

*Strain/Plasmid*	*Relevant characteristics*	*Reference/Source*
**Bacteria**		
*S. meliloti*		
1021	Wild-type SU47 derivative, Sm^r^	[75]
2011	Wild-type SU47 derivative, Sm^r^	[76]
1021Δ*hfq*	1021 *hfq *mutant strain, Sm^r^	This work
2011-1.2	2011 *hfq *insertion derivative (control str.), Sm^r^, Km^r^	This work
2011-3.4	2011 *hfq *insertion mutant, Sm^r^, Km^r^	This work
1021*hfq*^FLAG^	1021 derivative expressing a 3 × Flag-tagged Hfq, Sm^r^	This work
*E. coli*		
DH5α	F^- ^*endA1, glnV44, thi-1, recA1, relA1, gyrA96, deoR, nupG*, φ*80d, lacZ*ΔM15 Δ(*lacZYA-argF*)U169, *hsdR17*(r_K_^- ^m_K_^+^), λ-	Bethesda Research Lab.
**Plasmids**		
pRK2013	Helper plasmid, *Col*E1, Km^r^	[77]
pGEM^®^-T Easy	Cloning vector for PCR, Ap^r^	Promega Corporation
pK18*mobsacB*	Suicide vector in *S. meliloti *Km^r^, *sacB*, *ori*V	[78]
pJB3Tc19	Broad host-range IncP cloning vector, Ap^r^, Tc^r^	[79]
pBluescriptII KS+	Multicopy cloning vector, Ap^r^	Stratagene
pGEM*hfq*	1,684-bp of *hfq *genomic region in pGEM-T	This work
pK18_1.2	Internal fragment of *hfq *ORF in pK18*mobsacB*	This work
pK18_3.4	Internal fragment of *hfq *ORF in pK18*mobsacB*	This work
pGEMΔ*hfq*	Genomic region with deletion of *hfq *in pGEM-T	This work
pK18Δ*hfq*	Genomic region with deletion of *hfq *in pK18	This work
pGEMHfq	pGEM-T containing *hfq *promoter and ORF	This work
pJBHfq	pJB3Tc20 containing *hfq *promoter and ORF	This work
pKS3 × Flag	3 × FLAG encoding fragment in pBluescriptII KS+	This work
pKS3 × Flag5	pKS3 × Flag with upstream region of *hfq*	This work
pKSHfq3 × Flag	pBluescriptII KS+ with Flag-tagged *hfq *ORF	This work
pGEMHfq3 × Flag	pGEM-T with Flag-tagged *hfq *ORF	This work
pK18Hfq3 × Flag	pK18*mobsacB *with Flag-tagged *hfq *ORF	This work

Growth rates of rhizobial strains were monitored in an automated BioScreen C MBR machine (Growth Curves USA, Piscataway, NJ). Tested strains were initially grown in TY to late log phase (10^9 ^cells/ml; O.D_600 nm _0.9-1.0). Aliquots of 1 ml of the starting cultures were centrifuged; the pelleted cells were washed with fresh TY and finally resuspended in 1 ml of the medium. Ten μl of the bacterial suspensions (~10^7 ^cells) were inoculated into 340 μl of TY broth in Bioscreen Honey comb 100-well plates which were incubated at 30°C with continuous shaking. Absorbance readings at 600 nm were recorded every 2 h until the cultures reached the late stationary phase. OD values of uninoculated media were subtracted from cultures OD readings to normalize data for background prior to plotting. Determinations were done in triplicate for each strain.

### Construction of the *S. meliloti hfq* mutant derivatives

A 1,684-bp DNA region containing the 243-bp *hfq *ORF and flanking sequences (714-bp upstream and 727-bp downstream of *hfq*) was PCR amplified with *Pfu *polymerase using the primers pair Hfq_Fw/Hfq_Rv (for all the oligonucleotides cited hereafter see the additional file 3: oligonucleotide sequences) and *S. meliloti *1021 genomic DNA as template. This DNA fragment was inserted into pGEM^®^-T Easy vector generating plasmid pGEM*hfq*. For the construction of the *S. meliloti *2011 *hfq *insertion mutant derivatives two internal regions of the gene were *Taq *amplified from pGEM*hfq *with primers combinations hfqforw1/hfqrev2 and hfqforw3/hfqrev4 and subcloned into the suicide vector pK18*mobsacB *generating plasmids pK18_1.2 and pK18_3.4, respectively. Both plasmids were independently conjugated into the 2011 wild-type strain by triparental matings, using pRK2013 as helper, yielding mutants 2011-1.2 and 2011-3.4 which were selected as Km^r^Sm^r ^colonies in MM agar as a result of pK18*mobsacB *integration into the *hfq *gene by single homologous recombination events. Mutations were verified by PCR and the precise location of plasmid insertion into the *hfq *gene was determined by sequencing of the PCR products. For the generation of the *S. meliloti *1021 *hfq *deletion mutant, plasmid pGEM*hfq *was amplified with *Pfu *polymerase with divergent primers (hfqi_1/hfqi_2) flanking the *hfq *ORF and carrying an internal *Hind*III restriction site. The PCR product was *Hind*III-digested and autoligated generating plasmid pGEMΔ*hfq *that contains a 1,447-bp *S. meliloti *1021 genomic region in which the *hfq *ORF was deleted and replaced by a *Hind*III site. This region was retrieved from pGEMΔ*hfq *and inserted into pK18*mobsacB *as an *Eco*RI fragment yielding pK18Δ*hfq *which was mobilized into the wild-type 1021 by a triparental mating with pRK2013 as helper. Transconjugants arising from a single cross-over event were selected as Km^r^Sm^r ^colonies in MM and simultaneously verified to retain sacarose sensitivity in TY agar (10% sucrose). Km^r^Sm^r^Sac^s ^bacteria from an isolated colony were further cultured in TY broth and 10^6 ^cells from this culture were finally plated on TY agar containing 10% sucrose to select double cross-over events (i.e. excision of pK18*mobsacB*). Deletion of the *hfq *gene in the mutant bacteria was checked by colony PCR with oligonucleotides 5HfqMut/3HfqMut followed by *Hind*III restriction of the PCR products.

To express Hfq under the control of its own promoter for complementation of the mutants an 842-bp DNA fragment containing the Hfq coding sequence along with 571 nt of the upstream region was PCR amplified with *Pfu *using primers 5Hfq_C/3Hfq_C and pGEM*hfq *as the template. The PCR product was inserted into pGEM^®^-T Easy yielding pGEMHfq and finally cloned into the low copy plasmid vector pJB3Tc19 as an *Eco*RI fragment generating pJBHfq which was conjugated into the *S. meliloti hfq *mutant derivatives by triparental matings.

Modification of the chromosomal *hfq *gene to express a C-terminal epitope-tagged Hfq protein was done as follows. A dsDNA fragment encoding 3 tandem FLAG epitopes (3 × FLAG; Sigma-Aldrich) was first generated by annealing of the 3 × Flag and 3 × Flag-i 69mer oligonucleotides which were designed to leave 5'-end overhangs complementary to *Xba*I and *Hind*III recognition sequences. The resulting DNA fragment was then inserted between these two restriction sites in pBluescript II KS+ giving pKS3 × Flag. The full-length Hfq coding sequence (without the TGA stop codon) along with 655 bp of its upstream genomic region was PCR amplified from pGEM*hfq *with the primers pair 5HfqTag/3HfqTag both carrying *Xba*I sites at the 5'-end. The resulting PCR product was cloned into pGEM^®^-T Easy and retrieved as an *Xba*I DNA fragment which was gel purified and inserted at the *Xba*I site of pKS3 × Flag yielding pKS3 × Flag5. A second 873-bp DNA fragment containing the stop codon for the translation of the epitope-tagged Hfq protein was generated by PCR amplification of the *hfq *downstream region from pGEM*hfq *using the primers pair 5FlxTag/3FlxTag which incorporates *Hind*III sites at both ends of the resulting fragment. The amplification product was inserted into pGEM^®^-T Easy, recovered as a *Hind*III fragment, gel purified and finally cloned into the *Hind*III site of pKS3 × Flag5 to obtain pKSHfq3 × Flag. This plasmid was used as template to amplify an 1,839-bp DNA fragment with a variant of primers 5HfqTag and 3FlxTag in which the *Xba*I and *Hind*III sites were replaced by *Eco*RI and *Sph*I sites, respectively. The resulting PCR product was cloned in pGEM^®^-T Easy to obtain pGEMHfq3 × Flag, then retrieved as an *Eco*RI/*Hind*III fragment and finally inserted between these two restriction sites in the polylinker of pK18*mobsacB *giving pK18Hfq3 × Flag. This plasmid was mobilized by a triparental mating to the wild-type strain 1021 for replacement of the *hfq *gene by the modified allele. Four out of the 18 colonies screened by colony PCR after the second cross-over event were found to incorporate the 3 × FLAG coding sequence and were kept for further Western analysis with commercial FLAG antibodies (Sigma-Aldrich).

All plasmid constructs requiring previous PCR amplification of the cloned inserts were checked by sequencing. The correct genomic arrangements in all the *S. meliloti hfq *derivative strains were assessed by Southern hybridization of genomic DNA with the appropriate radioactive labeled dsDNA probes using standard protocols.

### Transcriptomics

Total rhizobial RNA was purified from log cultures in TY broth (10 ml) using the RNeasy Mini Kit (Qiagen, Hilden, Germany) following manufacturers instructions. Cy3- and Cy5-labeled cDNAs were prepared from 20 μg total RNA according to an amino-allyl dye coupling protocol as previously described [66,67].

Two slide (Sm14KOLI microarrays) hybridizations were performed with labeled cDNA from RNA preparations corresponding to 3 independent bacterial cultures following described protocols [67,68]. This represents a total of 12 potential hybridization data per spot. Slides were scanned with the GenePixTM Personal 4100A Microarray Scanner (MDS Analytical Technologies Inc., Sunnyvale, CA, USA). Mean hybridization signal and mean local background intensities were determined for each spot of the microarray images with the GenePix 5.0 software for spot detection, image segmentation and signal quantification (MDS Analytical Technologies Inc., Sunnyvale, CA, USA). The log_2 _value of the ratio of intensities was determined for each spot according to *M*_*i *_= log_2_(*R*_*i*_/*G*_*i*_), being *R*_*i *_= *I*_ch1*i *_- Bg_ch1*i *_and *G*_*i *_= I_ch2*i *_- B_gch2*i*_; where *I*_ch1*i *_and I_ch2*i *_are the signal intensities in channels 1 and 2, respectively, and Bg_ch1*i *_and B_gch2*i *_are the background intensities of each spot in channels 1 and 2, respectively. The mean intensity (*A*_*i*_) was calculated for each spot using the formula: *A*_*i *_= log_2_(*R*_*i*_*G*_*i*_)^0.5 ^[67]. Normalization and *t*-statistics were carried out with the EMMA 2.8.2 software developed at the Bioinformatics Resource Facility, Center for Biotechnology (CeBiTec), Bielefeld University (https://www.cebitec.uni-bielefeld.de/groups/brf/software/emma/cgi-bin/emma2.cgi[69]) which implements a normalization method based on local regression accounting for intensity and spatial dependence in dye biases [70]. Genes were scored as differentially expressed if the confidence indicator *P *was ≤ 0.05, the mean intensity *A *≥ 8 and the expression ratio M ≥ 1 or ≤ -1, as calculated from at least eight of the 12 replicates per spot.

### Proteomics

Preparation of protein extracts and 2D-gel electrophoresis were carried out essentially as described previously [71]. The *S. meliloti *wild-type 2011 and derivative strains 2011-1.2 and 2011-3.4 were grown in 500 ml TY broth to log phase (OD_600_: 0.5-0.9). Bacteria were harvested by centrifugation at 4°C and 6,000 × g for 20 min and cells were washed twice with LS buffer (68 mM NaCl, 3 mM KCl, 1.5 mM KH_2_PO_4_, 9 mM NaH_2_PO_4_). The pellet was resuspended in 5 ml of lysis buffer (40 mM Tris-HCl pH 8.5, 40 μg/ml RNase, 20 μg/ml DNase, 0.1 mM phenylmethylsulphonyl fluoride). The cells were disrupted by either sonication or French press. Cell debris were removed by centrifugation at 4°C and 12,000 × g for 20 min. Proteins were precipitated during 4 h with 4 volumes of cold acetone and collected by centrifugation at 4°C and 15,000 × g for 10 min. Acetone was allowed to evaporate in a laminar flow cabinet and the proteins were solubilized in free-dithiothreitol (DTT) rehydration solution (8 M urea, 2% CHAPS and traces of bromophenol blue). Protein concentration in the supernatant was determined by the Bradford assay. For 2D electrophoresis, 600 μg of proteins were solubilized in 495 μl of rehydration solution and 5 μl of 28% DTT and 2.5 μl of IPG buffer were added. The mixture was subjected to isoelectric focusing using Immobiline DryStrip (18 cm-pH 4 to 7) (Amersham Biosciences) using the following program: 1 h at 0 V, 12 h at 30 V, 2 h at 60 V, 1 h at 500 V, 1 h at 1000 V and a final phase of 8,000 V until reaching 75,000 V/h. The strips were equilibrated for 15 min with shaking in a solution of 50 mM Tris-HCl pH 8.8 containing 6 M urea, 30% glycerol, 2% SDS and 2% DTT, subjected to a second equilibration for 15 min with the same solution containing 2.5% iodoacetamide and 0.01% of bromophenol blue instead of DTT and then loaded onto 12.5% polyacrylamide gels. Second-dimension electrophoreses were performed at 20 W per gel, with a previous 30 min step at 4 W per gel. Gels were stained with Coomassie blue R. Spots corresponding to differentially accumulated proteins were excised from the gels, digested with trypsin and subjected to MALDI-TOF MS (Unidad de Proteómica, Parque Científico de Madrid). Peptide fragmentation and sequencing was only performed if necessary. Protein identification was done with the help of PRIAM application (http://www.priam.prabi.fr) and MASCOT program [72].

### Reverse transcriptase PCR

Total RNA of the wild-type 1021 and 1021Δ*hfq *deletion mutant strains grown under both oxic and microoxic conditions was isolated with the RNeasy Mini Kit (Qiagen, Hilden, Germany) following manufacturers instructions. Each RNA sample (5 μg) was reverse transcribed with the AMV reverse transcriptase (Roche Diagnostics, Germany) using random hexamers as primers in 10 μl reaction mixtures. cDNA preparations were diluted to 100 μl and 1 μl of each sample was subjected to 25 cycles of PCR amplification for the detection of NifA and FixK1/K2 transcripts with primer pairs nifAFw/nifARv and fixKFw/fixKRv, respectively. As the reference, the abundance of the 16S RNA was assessed by amplification of each cDNA with primers 16SFw/16SRv. Possible contamination of the RNA preparations with DNA was assessed by PCR amplification of the samples with each combination of primers.

### Plant assays and nodule microscopy

*Medicago sativa *L. 'Aragón' seeds were surface sterilized as previously described [73], germinated on 0.8% water agar plates in the dark at 28°C for 24 h, and finally transferred to either test tubes, Leonard assemblies or agar plates containing a nitrogen-free nutrient solution [74]. Seedlings were inoculated with 1 ml of a bacterial suspension at OD_600 nm _0.05. Nodulation kinetics of the assayed strains were determined in two independent sets of 24 plants grown hydroponically in test tubes by recording the number of nodulated plants and the number of nodules per plant at different days after inoculation. For competition assays, 7-days-old alfalfa plants grown in Leonard jars or agar plates were inoculated with 1:1 mixtures of the *S. meliloti *wild-type 2011 strain and its *hfq *insertion mutant derivative 2011-3.4 (Km^r^). A representative number of mature nodules (50-130 depending on the experiment) were collected 30 days after plants inoculation, surface-sterilized for 5 min in 0.25% HgCl_2_, crushed and simultaneously plated on TY and TY-Km agar to record the number of nodules invaded by wild-type and 2011-3.4 strains. The efficiency of the reference *S. meliloti *1021 and its *hfq *deletion mutant derivative (1021Δ*hfq*) in symbiotic nitrogen fixation was assessed in Leonard assemblies by determination of the dry weigh of individual plants 30 days after inoculation with the rhizobial strains.

Microscopy was performed on mature (30-days-old) nodules from plants grown and inoculated in agar plates. Nodulated roots were embedded in 3% agarose and 100 μm-transversal sections were made using a Leica VT1200S vibratome. Nodule sections were observed under an optical Nikon AZ100 microscope.

### Western blot and co-inmunoprecipitation assays

To verify the expression of the 3 × FLAG tagged Hfq protein, 0.05 OD whole cell protein fractions of the *S. meliloti *1021 wild-type strain and the *S. meliloti *1021*hfq*^FLAG ^derivatives (two independent clones arising from the second crossing-over were tested) were resolved by SDS-PAGE and transferred to nitrocellulose membranes by electroblotting during 50 min at 100 mA (TE77PWR semidry apparatus, Amersham Biosciences). Membranes were blocked for 1 h in 1.5% dry milk in TBS (20 mM Tris-HCl pH 8, 0.18 M NaCl) and hybridized as follows: ANTI-FLAG^® ^monoclonal antibody (Sigma #F7425; 1/1000 in TBS) for 1 h at room temperature, 3 × 10 min wash in TBS, α-mouse-HRP (1/5000 in TBS) for 1 h at room temperature, 3 × 10 min wash in TBS. Blots were developed by incubation for 2-3 min in 20 ml of luminol solution [50 mM Tris-HCl pH 8.6, NaCl 150 mM, 8 mg luminol (Sigma Aldrich), 1 mg 4-iodophenol, 0.01% H_2_O_2_] and exposed to Konica Minolta medical films.

For co-inmunoprecipitation, the 1021 wild-type strain and its derivative 1021*hfq*^FLAG ^were cultured in 200 ml of TY broth to OD_600 nm _0.6. Total RNA was prepared from 25 ml of each culture as previously described [30]. The remaining cells (175 ml) were collected by centrifugation (10 min, 4000 × g, 4°C). The pellets were washed with cold PBS, chilled on ice, resuspended in 8 ml of lysis buffer (50 mM Tris-HCl, pH 7.4, 150 mM NaCl, 1 mM EDTA, and 1% TRITON X-100) and disrupted by sonication in three cycles of 10 s bursts at 32 W with a microprobe. Cell lysates were incubated 30 min at 4°C with shaking and centrifuged (20 min, 12000 × g, 4°C). Forty microliters of the ANTI-FLAG M2^® ^resin (Sigma #A2220) were added to the cleared lysates followed by incubation overnight with shaking at 4°C. The suspensions were centrifuged; the beads were resuspended in 1 ml lysis buffer and transferred to spin columns, followed by five washes in 1 ml of the same buffer. Protein/RNA complexes were recovered from beads by incubation with 15 ng of 3 × FLAG Peptide^® ^(Sigma #F4799) followed by elution in 100 μl of water. Phenol:chloroform extracted RNA was concentrated by ethanol precipitation and resuspended in 70 μl of water. Aliquots of 10 μg of total RNA and 10 μl of the co-inmunoprecipitated RNA were subjected to Northern analysis with the Smr sRNAs probes as described [30].

## Authors' contributions

OT-Q carried out transcriptomics, nodulation tests/microscopy of the 1021Δ*hfq *mutant and CoIP experiments; RIO, performed proteomics of the 2011-3.4 mutant and competition tests, and contributed to the design of the study; AP, performed RT-PCR experiments; EJ, contributed to the proteomic profiling; JL, contributed to the analysis of proteomic data and revised the manuscript; RR, contributed to the design of the study, analyzed data and critically revised the manuscript; NT, revised the manuscript; JIJZ, conceived and designed the study, obtained 1021Δ*hfq *and 1021*hfq*^FLAG ^strains and wrote the paper. All authors read and approved the final manuscript.

## Supplementary Material

Additional file 1 **Differentially accumulated transcripts in *S. meliloti *1021 and 1021Δ*hfq *derivative strain**. List of down- and up-regulated genes grouped by functional categories according to the *S. meliloti *genome database and KEGG. Click here for file

Additional file 2 **Differentially accumulated proteins in *S. meliloti *2011 wild-type and 2011-3.4 insertion mutant derivative**. List of down- and up-regulated proteins and their adscription to functional categories according to the *S. meliloti *genome database and KEGG. Click here for file

Additional file 3 **Oligonucleotide sequences**. Sequences of the oligonucleotides used in this study. Click here for file
