# Supplementary material for: The Sinorhizobium meliloti RNA chaperone Hfq influences central carbon metabolism and the symbiotic interaction with alfalfa
Source: BMC Microbiol. 2010 Mar 6;10:71. doi: 10.1186/1471-2180-10-71 (PMC2848018; doi:10.1186/1471-2180-10-71)
Supplement: Additional file 1 — Differentially accumulated transcripts in S. meliloti 1021 and 1021Δhfq derivative strain. List of down- and up-regulated genes grouped by functional categories according to the S. meliloti genome database and KEGG. [file 1471-2180-10-71-S1.PDF]

Down-regulated transcrips in the 1021Δ*hfq* mutant

	Putative function	M value
TRANSPORT		
ABC transporter myo-inositol		
*ibpA (SMb20712)**	myo-inositol transporter/periplasmic binding protein	-1,24
iatA (SMb20713)	ATP-binding protein	-2,82
iatP (SMb20714)	Inner membrane component/Permease	-1,05
ABC transporter α-glucosides/carbohydrates/organic acids/alcohols		
*aglE (SMc03061)**	Periplasmic solute-binding protein	-3,94
aglF (SMc03062)	Inner membrane component/Permease	-1,21
Other transport genes		
SMc02514	ABC transporter glycerol-3-phosphate and glycerol/periplasmic solute-binding component	-2,05
SMc02519	Putative glycerol-3-phosphate and glycerol ABC transporter/ATP-binding component	-2,85
*frcB (SMc02171)**	Putative fructose ABC-type transport system/periplasmic component	-1,04
SMc02031	Ribose transport system permease	-1,29
SMc04317	ABC transporter/periplasmic solute-binding component/Fe uptake	-1,04
SMc04439	Putative ABC-transporter ATP-binding protein/histidine, glycine betaine	-1
SMb20072	ABC transporter/sugars/periplasmic solute-binding protein, induced by myo-inositol	-5,87
SMb20144	ABC transporter/periplasmic solute-binding protein/carbohydrates, org. acids, alcohols	-1,27
SMALL MOLECULE METABOLISM		
myo-inositol catabolism operon		
iolC (SMc01165)	Putative sugar kinase	-1,2
*iolD (SMc01166)**	Putative malonic semialdehyde oxidative decarboxylase	-2,01
*iolE (SMc00433)**	Putative myo-inositol catabolism protein/Xylose isomerase-like, TIM barrel	-1,79
iolB (SMc00432)	Putative myo-inositol catabolism protein/Cupin, RmlC-type	-1,67
Other genes related to small molecule metabolism		
SMc01163	Glucose-fructose oxidoreductase	-1,48
ilvC (SMc04346)	Ketol-acid reductoisomerase/branched-chain amino acid biosynthesis	-1,83

SMc03211	Putative 4-hydroxyphenylpyruvate dioxygenase/aromatic amino acids metabolic process	-2,48
SMc03253	L-proline cis-4-hydroxylase/peptidyl-amino acid modification	-2,09
pheAa (SMc03858)	Putative chorismate mutase/aromatic amino acids biosynthetic process	-1,18
mtbC (SMc04331)	Putative dimethylamine corrinoid protein/methionine biosynthetic process	-2,16
cobP (SMc04305)	Bifunctional adenosylcobalamin biosynthesis protein CobP	-1,41
SMc04342	Dihydropteroate synthase (DHPS)/folic acid and derivative biosynthetic process	-1,61
mttB1 (SMc04330)	Putative trimethylamine methyltransferase/methanogenesis	-1,97
SMc02045	D-3-phosphoglycerate dehydrogenase/Acetylation Amino-acid biosynthesis	-1,05
bkdAb (SMc03202)	Probable 2-oxoisovalerate dehydrogenase beta subunit/glycolysis oxidoreductase	-1,04
SMc02689	Probable aldehyde dehydrogenase	-1,12
pyrC (SMc02166)	Probable dihydroorotase/pyrimidine base biosynthetic process	-1,18
glyA1 (SMc01770)	Probable serine hydroxymethyltransferase/glycine metabolic process	-1,22
SMc00982	Putative dioxygenase Rieske 2Fe-2S family protein/carbon compounds	-1,13
purU1 (SMc03205)	Putative formyltetrahydrofolate deformylase/purine ribonucleotide biosynthesis	-2,71
soxB2 (SMc03933)	Putative sarcosine oxidase subunit B/tetrahydrofolate metabolic process	-2,65
frcK (SMc02167)	Putative fructose transport system kinase	-1,36
adhA1 (SMa1296)	Alcohol dehydrogenase, Zn-dependent class III/fermentation	-3,24
SMa1156	Alcohol dehydrogenase, Zn-dependent class III/Energy metabolism, carbon	-1,93
hemN (SMa1266)	HemN coproporphyrinogen III oxidase/porphyrin biosynthetic process	-2,27
SMa1166	Protein containing an alpha/beta hydrolase fold/alpha-beta hydrolase	-1,15
azu1 (SMa1243)	Azu1 pseudoazurin	-1,71

OTHER CELL PROCESSES

Electron transport/Nitrogen fixation

Operon fixN1-O1-Q1-P1-G-H-I1-S1		
fixN1 (SMa1220)	FixN1 cytochrome c oxidase subunit 1/Cytochrome c oxidase, subunit I	-1,27
fixQ1 (SMa1214)	FixQ1 nitrogen fixation protein/Cbb3-type cytochrome oxidase component	-1,78
fixP1 (SMa1213)	FixP1 di-heme c-type cytochrome/Cytochrome c oxidase cbb3-type, subunit III	-1,2
fixG (SMa1211)	FixG iron sulfur membrane protein/Cytochrome c oxidase cbb3 type, accessory protein FixG	-1,15
Operon fixK2-N2-O2-Q2-P2		
fixK2 (SMa0762)	FixK2 transcriptional regulator	-2,42
SMa0763	FixM flavoprotein oxidoreductase	-2,49
fixQ2 (SMa0767)	FixQ2 nitrogen fixation protein/Cbb3-type cytochrome oxidase component	-1,96
Operon fixL-J-T1-K1-M		

fixK1 (SMa1225)	FixK1 transcriptional regulator	-1,7
fixM (SMa1223)	FixM flavoprotein oxidoreductase	-2,86
SMa1151	Putative FixM flavoprotein oxidoreductase	-1,96
napF (SMa1240)	NapF component of periplasmic nitrate reductase/anaerobic respiration	-1,82
SMa1170	Probable Cytochrome c	-1,29
cycA (SMb21367)	Putative cytochrome c class I protein, probably cytochrome c4	-1,65
etfA1 (SMc00728)	Putative electron transfer flavoprotein alpha-subunit alpha-ETF flavoprotein	-1,01
ccmD (SMc03850)	Putative heme exporter D (cytochrome C-type biogenesis protein) transmembrane	-1,34
Central intermediary metabolism		
phoX (SMc02634)	Monomeric alkaline phosphatase/phosphorus metabolism	-2,03
SMc02148	Putative polyphosphate kinase 2	-1,27
Operon nosRZDFYLX		
nosZ (SMa1182)	NosZ nitrous oxide reductase/nitrogen metabolism	-1,39
nosD (SMa1183)	NosD nitrous oxidase accessory protein/nitrogen metabolism	-2,13
Macromolecule metabolism		
SMa1126	Putative protease, transmembrane protein	-2,8
degP4 (SMa1128)	DegP4 protease like protein	-1,03
Transcriptional regulators		
SMc02030	Transcription regulator LysR family	-1,08
fixT3 (SMc03254)	Putative antikinase	-1,09
nrdR (SMc01771)	Putative transcriptional repressor	-1,07
SMa1207	Transcriptional regulator, CAP/Crp family	-1,53
Miscellaneous		
flaA (SMc03037)	Flagellin A/Mobility chemotaxis	-1,05
SMb21053	Putative membrane-anchored glycosyltransferase/surface polysaccharides biosynthesis	-1,35
cyaP (SMa1103)	Adenylate/guanylate cyclase	-2,01
SMa1149	Universal stress protein (Usp)/response to stress	-2,76
SMa1147	Universal stress protein (Usp)/response to stress	-2,55
SMa1158	Universal stress protein (Usp)/response to stress	-1,8

HYPOTHETICAL PROTEINS

Putative transmembrane proteins

SMc01986	Hypothetical transmembrane protein	-1,97
SMc00317	Putative transporter/auxin efflux carrier	-1,89
SMb20139	Conserved hypothetical transmembrane protein	-1,66
SMa1097	Hypothetical transmembrane protein	-1,41

Other proteins

SMc04339	Hypothetical protein	-2,52
SMc04310	Hypothetical protein	-1,9
SMc01747	Hypothetical protein	-1,78
SMc04345	Hypothetical protein	-1,67
SMc00391	Hypothetical protein	-1,34
SMc01600	Conserved hypothetical protein	-1,19
SMc00472	Conserved hypothetical protein	-1,14
SMc04337	Conserved hypothetical protein	-1,06
SMc03251	Conserved hypothetical protein	-1,05
SMa1082	Hypothetical protein	-2,13
SMa1154	Conserved hypothetical protein	-2
SMa1050	Hypothetical protein	-1,45
SMa0633	Hypothetical protein	-1,29
SMa1176	Hypothetical protein	-1,24
SMa1077	Nex18 Symbiotically induced conserved protein	-1,24
SMa1052	Conserved hypothetical protein	-1,02

Up-regulated transcrips in the 1021Δ*hfq* mutant

TRANSPORT

ABC Transporter L-aminoacids/Peptides

*aapJ (SMc02118)**	Extracellular solute-binding protein	1,7
aapQ (SMc02119)	Inner membrane component	1,42
*aapP (SMc02121)**	ATP-binding protein	1,95

ABC Transporter Spermidine/putrescine		
SMc01966	Extracellular solute-binding protein	1,06
SMc01965	ATP-binding protein	6,39
SMc01963	Permease	1,56
High-affinity branched-chain amino acid transport		
livH (SMc01951)	Inner-membrane translocator	4,99
livM (SMc01950)	Inner-membrane translocator	1,6
livG (SMc01949)	ATP-binding protein	1,91
livF (SMc01948)	ATP-binding protein	2,55
SMc01947	Conserved hypothetical transmembrane protein	3,39
*livK (SMc01946)**	Leu/Ile/Val-binding protein	1,88
ABC Transporter Uracil/uridine		
SMc01823	ATP-binding protein	1,74
SMc01824	Conserved hypothetical protein/GCN5-related N-acetyltransferase	1,07
SMc01825	Inner membrane component	1
SMc01827	Periplasmic solute-binding protein	2,99
ABC Transporter Putrescine/agmatine		
SMc01652	Solute-binding protein	1,04
SMc01653	ATP-binding protein	1,26
ABC Transporter Tetrapeptides/tripeptides		
oppB (SMb21197)	Inner membrane component/Permease	1,98
oppC (SMb21198)	Inner membrane component/Permease	1,82
Other transport genes		
SMc02603	Permease	1,02
SMc00140	Putative amino-acid binding periplasmic protein	2,35
SMc04127	ATP-binding protein/Not classified ABC transporter	1,31
SMc01597	Putative amino-acid permease	1,27
*SMc02259**	Putative periplasmic binding ABC transporter	1,55
lolC (SMc01935)	Lipoprotein-releasing system transmembrane protein/transport of large molecules	1,9

SMb21572	ABC transporter/periplasmic solute-binding protein precursor/amino acids	1,44
SMb20605	ABC transporter/periplasmic solute-binding protein	1,07
SMb21151	Putative sugar amine ABC transporter/periplasmic solute-binding protein	1,23
SMb20504	Putative ABC transporter periplasmic sugar-binding protein	1,45
SMb20442	Put. Trap-type pentose monosaccharide transp. system/peripl. solute-binding protein	1,03
SMc01962	Conserved hypothetical protein/nitrogen compound metabolic process acting on C-N	1,45
SMc01961	Conserved hypothetical protein/bleomycin resistance protein/dioxygenase	2,19
SMc01960	NADPH-dependent FMN reductase	2,35

SMALL MOLECULE METABOLISM

Glycine cleavage system

gcvP (SMc02049)	Glycine dehydrogenase	2,06
gcvH (SMc02048)	Glycine cleavage H-protein	2,02
gcvT (SMc02047)	Glycine cleavage T-protein	3,32

Other genes related to small molecule metabolism

SMc01930	Putative methylmalonyl-CoA epimerase	3,26
SMc01815	Dihydropyrimidine dehydrogenase	2,21
SMc00115	Putative glyoxalase/bleomycin resistance protein/dioxygenase	1,35
SMc03773	Putative acetyltransferase	1,21
cyaF7 (SMb20300)	Putative adenylate cyclase/global regulatory functions	1
SMb21202	Glyoxalase/bleomycin resistance protein/dioxygenase	1,09
SMa0374	Glyoxalase/bleomycin resistance protein/dioxygenase	1,06
SMa0376	Isochorismatase hydrolase/Cysteine hydrolase superfamily	1,7
SMa1038	Multicopper oxidase	1,14
SMa0380	Putative hydrolase/phosphonate metabolism	1,58
SMa1041	Copper binding protein/electron transport	1,32

OTHER CELL PROCESSES

Transcriptional regulators

ohrR (SMc01945)	MarR transcriptional regulator (detoxification)	1,87

Macromolecule metabolism

miaA (SMc01435)	Probable tRNA delta2-isopentenylpyrophosphate transferase/tRNA modification	1,02
degP1 (SMc02365)	Probable serine protease/degradation of proteins, peptides, glycopeptides	1,11
SMc00414	Putative phospholipid N-methyltransferase	1,69
SMc01929	RNaseJ	2,96
SMc03796	Endoribonuclease L-PSP/Cleaves mRNAs and inhibits traslation	1,56
SMa0426	Putative ATP-dependent DNA-helicase	1,07
Central intermediary metabolism		
SMc01814	Probable glutamate synthase small chain/central intermediary metabolism	2,33
dht (SMc01821)	Putative D-hydantoinase (dihydropyrimidinase)/central intermediary metabolism	1,1
xdhA2 (SMb21011)	Xanthine dehydrogenase/electron transport	1,46
Structural elements		
rplS (SMc03863)	50S Ribosomal protein	1,09
rpmA (SMc03772)	50S Ribosomal protein L27	2,2
Miscellaneous		
gst8 (SMc03882)	Putative glutathione S-transferase	1,03
wrbA1 (SMc00943)	Flavoprotein WrbA	1,1
wrbA3 (SMa1935)	Flavoprotein WrbA	1,24
cvpA (SMc00555)	Putative colicin V production homolog transmembrane protein	1,11
SMc04451	Putative chloramphenicol 3-O phosphotransferase/Drug-analog sensitivity and resistance	1,99
SMb20534	Xylose isomerase-like protein	1,3
HYPOTHETICAL PROTEINS		
Putative transmembrane proteins		
SMc01931	Conserved hypothetical transmembrane protein	2,59
SMc01763	Hypothetical transmembrane protein	1,08
SMc02174	Hypothetical transmembrane protein	1,06
SMb20724	Conserved hypothetical exported protein	1,01
SMa1507	Conserved hypothetical protein/basic membrane lipoprotein	1,06
Other proteins		
SMc01957	Conserved hypothetical protein	2,43

SMc00617	Conserved hypothetical protein	1,07
SMc04164	Conserved hypothetical protein	1,03
SMa0592	Conserved hypothetical protein	1,61
SMa0674	Hypothetical protein	1,55
SMa1043	Conserved hypothetical protein	1,41

Note: **Genes also identified by the proteomics analysis*
