# Supplementary material for: The Sinorhizobium meliloti RNA chaperone Hfq influences central carbon metabolism and the symbiotic interaction with alfalfa
Source: BMC Microbiol. 2010 Mar 6;10:71. doi: 10.1186/1471-2180-10-71 (PMC2848018; doi:10.1186/1471-2180-10-71)
Supplement: Additional file 2 — Differentially accumulated proteins in S. meliloti 2011 wild-type and 2011-3.4 insertion mutant derivative. List of down- and up-regulated proteins and their adscription to functional categories according to the S. meliloti genome database and KEGG. [file 1471-2180-10-71-S2.PDF]

Table 1. Differentially accumulated proteins in *S. meliloti* 2011 wild-type and 2011-3.4 *hfq* insertion mutant derivative as revealed by 2D gel analyses.

Candidate protein[a]	Function[c]
**GroEL2 (SMa0744)**	Chaperonin Cpn60/TCP-1
**GrpE (SMc01142)**	Probable heat shock protein/ GrpE nucleotide exchange factor
**IbpA (SMc04040)**	Probable heat shock protein/HSP20-like chaperone
**IbpA (SMb20712)***	Putative myo-inositol ABC transporter
**AraA (SMb20895)**	ABC transporter, sugar uptake
**FrcB (SMc02171)***	Putative fructose ABC transporter
**AglE (SMc03061)***	ABC transporter, α-glucosides
**IolE (SMc00433)***	Putative myo-inositol catabolism protein
**IolD (SMc01166)***	Putative malonic semialdehyde oxidative decarboxylase
**AcsA1 (SMc04093)**	Probable acetyl-coenzyme A synthetase
**Tig (SMc02050)**	Probable trigger factor
**SMc01242**	Conserved hypothetical signal peptide protein
GroEL5 (SMb21566)	Chaperonin Cpn60/TCP-1/Putative heat sock protein
SMc02259*	Putative amino acid ABC transporter
DppA1 (SMc00786)	Putative amino acid or peptide ABC transporter
DppA2 (SMc01525)	Putative dipeptide binding periplasmic protein
LivK (SMc01946)*	Putative leucine-specific binding protein precursor
AapJ (SMc02118)*	Probable general L-amino acid ABC transporter
AapP (SMc02121)*	Probable general L-amino acid ABC transporter
SMc02378	Putative glycine betaine ABC transporter
ChoX (SMc02737)	Putative choline ABC transporter
Ocd (SMb21494)	Putative ornithine cyclodeaminase
ArgI1 (SMc03091)	Probable arginase
PckA (SMc02562)	Phosphoenolpyruvate carboxykinase
AhcY (SMc02755)	Probable adenosylhomocysteinase
SMc04385	Putative aldehyde dehydrogenase transmembrane protein
SMc00242	Hypothetical signal peptide protein
Efp (SMc00357)	Probable elongation factor P
SMc02884	Probable membrane lipoprotein
SMc00777	Conserved hypothetical protein
SMc02354	Conserved hypothetical protein
SMc02503	Conserved hypothetical protein
SMc02911	Conserved hypothetical protein

[a] Nomenclature according to http://iant.toulouse.inra.fr/bacteria/annotation/cgi/rhime.cgi Down-regulated proteins in the mutant are in bold.
[b] Functional classification according to KEGG (http://www.genome.jp/kegg/).
*The corresponding coding transcripts were also found to be up/downregulated in the *S. meliloti* 1021Δ*hfq* mutant.
