# Supplementary material for: The Sinorhizobium meliloti RNA chaperone Hfq influences central carbon metabolism and the symbiotic interaction with alfalfa
Source: BMC Microbiol. 2010 Mar 6;10:71. doi: 10.1186/1471-2180-10-71 (PMC2848018; doi:10.1186/1471-2180-10-71)
Supplement: Additional file 3 — Oligonucleotide sequences. Sequences of the oligonucleotides used in this study. [file 1471-2180-10-71-S3.PDF]

Additional file 3. Oligonucleotide sequences

Name	Nucleotide sequence
Hfq_Fw	5’-TCCGCAACGGGCTCTTCTAT-3’
Hfq_Rv	5’-TGCCCGCGTTCGTATAGCCT-3’
hfqforw1	5’-AAAAGAATTCATCTCTTTCTCAACACGGTCC-3’
hfqrev2	5’-AAAAGGTACCCAGCTTCCTCATTCTCGAACA-3’
hfqforw3	5’-AAAAGAATTCGTCCGCAAGCAAAAGATTTC-3’
hfqrev4	5’-AAAAGGTACCCGTGCTTGTAGACGAGCTGA-3’
hfqi_1	5’-GTGTGTAAGCTTCGCGCCGCTTCTTTCTTA-3’
hfqi_2	5’-CCGTGTAAGCTTCGGGATGTGAGGAACAT-3’
5HfqMut	5’-TCTTCATCACCGCTGCTACC-3’;
3HfqMut	5’-AACGATCATGCCGTGAACGA-3’
5Hfq_C	5’-GGCATATCGGCGATAACG-3’
3Hfq_C	5’-CACGCTTGGTAATGTTCC-3’
3xFlag	5’-CTAGAGATTACAAAGACCATGACGGTGATTATAAA GATCATGACATCGACTACAAGGATGACGATGACA-3’
3xFlag-i	5’-AGCTTGTCATCGTCATCCTTGTAGTCGATGTCATG ATCTTTATAATCACCGTCATGGTCTTTGTAATCT-3’
5HfqTag	5’-TCTAGATTTCCCTGATGCGGACAC-3’
3HfqTag	5’-TCTAGAGGCAGCTTCCTCATTCTC-3’
5FlxTag	5’-AAGCTTTGACGGGATGTGAGGAAC-3’
3FlxTag	5’-AAGCTTAAATGAAGCCGACGGTGT-3’
nifAFw	5’-TCGTCTTGAGACCACGCTTA-3’
nifARv	5’-CATGACTTGGTCTATTGCGG-3’
fixKFw	5’-TCCATCGAGGTCGAACACCT-3’
fixKRv	5’-CATTTCGCCTGGGAGATGAA-3’
16SFw	5’-GGCTAGCGTTGTTCGGAATT-3’
16SRv	5’-TCCGATCCAGCCGAACTGAA-3’
